# Slowness and Sparseness Lead to Place, Head-Direction, and Spatial-View Cells

**DOI:** 10.1371/journal.pcbi.0030166

**Published:** 2007-08-31

**Authors:** Mathias Franzius, Henning Sprekeler, Laurenz Wiskott

**Affiliations:** Institute for Theoretical Biology, Humboldt-Universität zu Berlin, Berlin, Germany; University College London, United Kingdom

## Abstract

We present a model for the self-organized formation of place cells, head-direction cells, and spatial-view cells in the hippocampal formation based on unsupervised learning on quasi-natural visual stimuli. The model comprises a hierarchy of Slow Feature Analysis (SFA) nodes, which were recently shown to reproduce many properties of complex cells in the early visual system [1]. The system extracts a distributed grid-like representation of position and orientation, which is transcoded into a localized place-field, head-direction, or view representation, by sparse coding. The type of cells that develops depends solely on the relevant input statistics, i.e., the movement pattern of the simulated animal. The numerical simulations are complemented by a mathematical analysis that allows us to accurately predict the output of the top SFA layer.

## Author Summary

Rats excel at navigating through complex environments. In order to find their way, they need to answer two basic questions. Where am I? In which direction am I heading? As the brain has no direct access to information about its position in space, it has to rely on sensory signals—from eyes and ears for example—to answer these questions. Information about its position and orientation is typically present in the information it gathers from its senses, but unfortunately it is encoded in a way that is not obvious to decode. Three major types of cells in the brain whose firing directly reflects spatial information are place, head-direction, and view cells. Place cells, for example, fire when the animal is at a particular location independent of the direction the animal is looking in. In this study, we present a self-organizational model that develops these three representation types by learning on naturalistic videos mimicking the visual input of a rat. Although the model works on complex visual stimuli, a rigorous mathematical description of the system is given as well.

## Introduction

The brain needs to extract behaviorally relevant information from sensory inputs in order to successfully interact with the environment. Position and head direction of an animal in the space surrounding it is part of this relevant information. Neural representations of a rodent's spatial position—termed *place cells*—were found more than 35 years ago in hippocampal areas CA1 and CA3 [2], correlates of head orientation—termed *head-direction cells*—were found twenty years later [3], and recently nonlocalized representations—termed *grid cells—*were found in entorhinal cortex (EC) of rats [4]. Primates possibly also have place cells, certainly head-direction cells, and also *spatial-view cells* that do not encode the animal's own (idiothetic) position but fire whenever the animal views a certain part of the environment [5–8]. Grid cells in primates have not yet been reported.

**Figure 1:**
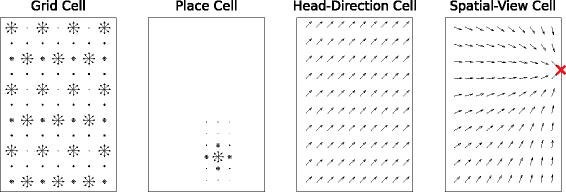
Spatial and Orientation Tuning of an Idealized Grid Cell, Place Cell, Head-Direction Cell, and a Spatial-View Cell Oriospatial activity is indicated by arrows. Length of arrows indicates strength of activity at the arrow base if the animal looks in the direction of the arrow. The activity of a grid cell is mostly orientation invariant and not spatially localized but repeats in a hexagonal grid, whereas a place cell is also orientation invariant but spatially localized. The activity of a head-direction cell shows a global direction preference but is spatially invariant, and the spatial-view cell is maximally active when a specific view is fixated (indicated by ×) with an amplitude that is independent of spatial position.

All of these cells selectively encode some aspects of position and/or orientation of the animal, while being invariant to others. Head-direction cells are strongly selective for the direction of the animal's head and largely invariant to its position [9]. They typically have a single peak of activity with a Gaussian or triangular shape and a tuning width of roughly 60° to 150° [10], depending on brain area. In contrast, most place cells recorded in open fields are invariant to head direction while being selective for the animal's position. Interestingly, the degree of orientation–invariance depends on the behavioral task of the animal and possibly on the structure of the environment. In linear track environments and for repeated linear paths in open environment most place cells are orientation-specific [11]. Grid cells in EC also exhibit conjunctive representations of position and orientation [12]. Spatial-view cells in primates show very different firing properties. These cells are neither position invariant nor orientation invariant but fire when a certain part of the environment is in the animal's field of view (FOV), resembling head-direction cells for the case of an infinitely distant view. Figure 1 illustrates the difference between grid cells, place cells, head-direction cells, and spatial-view cells.

Throughout this paper, *oriospatial cells* will be used as a superordinate term for place cells, grid cells, head-direction cells, and spatial-view cells. While the precise role of these oriospatial cells is still discussed, they probably form the neural basis for the ability of an animal to self-localize and navigate [13].

Stimuli available to oriospatial cells can be classified as either *idiothetic,* including motor feedback, proprioception, and vestibular input, or as *allothetic,* which includes all information from sensors about the external environment, e.g., vision or olfaction. While place cells are influenced by several modalities, they seem to be driven primarily by visual input (e.g., [14]), but since their firing properties remain stable in the absence of external sensory cues for several minutes, idiothetic stimuli must play a major role for place-cell firing as well [15]. Using idiothetic information for navigation, which is referred to as *path integration* (or dead reckoning), inherently accumulates errors over longer timescales, which can only be corrected by allothetic information. For the head-direction cells it is commonly assumed that idiothetic input from the vestibular system is dominant (e.g., [9]), but like place cells they need external sensory stimuli to correct for drift.

We introduce here a model for the self-organized formation of hippocampal place cells, head-direction cells, and spatial-view cells based on unsupervised learning on quasi-natural visual stimuli. Our model has no form of memory and receives raw high-dimensional visual input. The former means that our model cannot perform path integration, the latter means that positional information has to be extracted from complex images. While such a model can certainly not be a complete model of oriospatial cells, it can show how far a memoryless purely sensory-driven system can model oriospatial cells. The learning rule of the model is based on the concept of slowness or temporal stability, which is motivated by the observation that raw sensory signals (like individual pixel values of a camera) typically vary much more quickly than some behaviorally relevant features of the animal or its environment, such as the animal's position in space. By extracting slowly varying features from the sensory input, one can hope to obtain a useful representation of the environment. This *slowness principle* forms the basis for a variety of learning rules (e.g., [16–18]). The implementation used here is *Slow Feature Analysis* (SFA) as introduced by Wiskott [19,20]. For a given set of time-dependent training data, in our case video sequences, we are looking for a nonlinear scalar function from a given function space that generates the slowest possible output signal *y*(*t*) when applied to the training data. The slowness of the signal is measured in terms of its Δ-value, which is given by the mean square of the signal's temporal derivative (see the section Slow Feature Analysis). As small Δ-values correspond to slowly varying signals, the objective is to find the function that minimizes the Δ-value. To avoid the trivial constant solution, the signal is required to have unit variance and zero mean. Furthermore, we can find a second function that optimizes the objective under the additional constraint that its output signal is uncorrelated to the first, a third function, whose output is uncorrelated to the first two signals, and so on. In this manner we generate a sequence of functions with increasing Δ-value that extracts slowly varying features from the training data. More details on the approach as well as its mathematical formalization can be found in the section Slow Feature Analysis. It is important, however, to stress that SFA is not related to low-pass filtering, as the apparent paradox of slowly varying but instantaneously extracted output signals is a frequent source of misunderstandings. Low-pass filtering is a trivial way to generate slowly varying, but most often completely uninformative, outputs. Such signals cannot be instantaneous, as by definition they are generated by averaging over the past. In contrast, the representations our model finds depend on the temporal structure of sensory data *during the training phase* of the model, but once they are established they are instantaneous, i.e., a single “snapshot”of sensory stimuli is sufficient to generate the model output (e.g., a model place cell response).

SFA has been successfully applied as a model for the self-organized formation of complex cell receptive fields in primary visual cortex [1]. Here, we embed this approach in a biologically inspired hierarchical network of visual processing of a simulated rat where each layer learns the slowest features from the previous layer by SFA (see the section Experimental Methods). We find that the output of the highest layer performing SFA forms a distributed oriospatial representation. In a subsequent linear step, the model applies a mechanism for sparse coding resulting in localized oriospatial codes. The same model in the same environment can reproduce the firing characteristics of place cells, head-direction cells, and spatial-view cells, depending solely on the movement statistics of the simulated rat. For roughly uncorrelated head direction and body movement, the system learns head-direction cells or place cells depending on the relative speed of head rotation and body movement. If the movement statistics is altered such that spots in the room are fixated for a while during simulated locomotion, the model learns spatial-view cell characteristics.

Any computation in the brain is useless unless it leads to a change of behavior of the animal. We assume a phenomenological approach and model rat and primate oriospatial cells without asking the question what behavioral purpose these oriospatial cells serve. The last linear step of sparsification might seem irrelevant in this context; however, sparse codes have a number of advantages for subsequent processing steps that include easier decoding, energy efficiency, and, notably in the context of hippocampus, increased efficiency of memory storage in recurrent networks such as CA3 [21].

We introduce a mathematical framework in the section Theoretical Methods that analytically explains the results of the SFA output. The mathematically less inclined reader may consider skipping this section. Both analytical and computer simulation results are presented in the Results section.

We conclude that a purely sensory-driven model can capture the key properties of several major cell types associated with spatial coding, namely place cells, head-direction cells, spatial-view cells, and to some extent grid cells.

## Methods

### Slow Feature Analysis

SFA solves the following learning task: given a multidimensional input signal we want to find instantaneous scalar input–output functions that generate output signals that vary as slowly as possible but still carry significant information. To ensure the latter, we require the output signals to be uncorrelated and to have unit variance. In mathematical terms, this can be stated as follows.

***Optimization problem.*** *Given a function space ℱ and an I*-*dimensional input signal* **x**(*t*)*, find a set of* *J* *real-valued input–output functions g_j_*(**x**) ∈ ℱ *such that the output signals y_j_*(*t*) := *g_j_*(**x**(*t*))

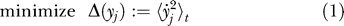

*under the constraints*

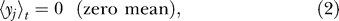


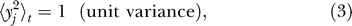


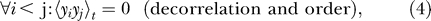

*with* 〈·〉*_t_ and* 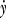 *indicating temporal averaging and the derivative of y, respectively.*

Equation 1 introduces the Δ-value, which is a measure of the temporal slowness of the signal *y*_*j*_(*t*). It is given by the mean square of the signal's temporal derivative, so small Δ-values indicate slowly varying signals. The constraints (2) and (3) avoid the trivial constant solution and constraint (4) ensures that different functions *g_j_* code for different aspects of the input.

It is important to note that although the objective is slowness, the functions *g_j_* are instantaneous functions of the input, so that slowness cannot be enforced by low-pass filtering. Slow output signals can only be obtained if the input signal contains slowly varying features that can be extracted instantaneously by the functions *g_j_*.

In the computationally relevant case where ℱ is finite-dimensional, the solution to the optimization problem can be found by means of SFA [1,20]. This algorithm, which is based on an eigenvector approach, is guaranteed to find the global optimum. Biologically more plausible learning rules for the optimization problem, both for graded response and spiking units, exist [22,23].

If the function space is infinite-dimensional, the problem requires variational calculus and will in general be difficult to solve. In the section The modified optimization problem, we demonstrate that the optimization problem for the high-dimensional visual input, as faced by the hierarchical model, can be reformulated for the low-dimensional configural input of position and orientation. In this case, the variational calculus approach becomes tractable and allows us to make analytical predictions for the behavior of the full model.

### Experimental Methods

**Figure 2:**
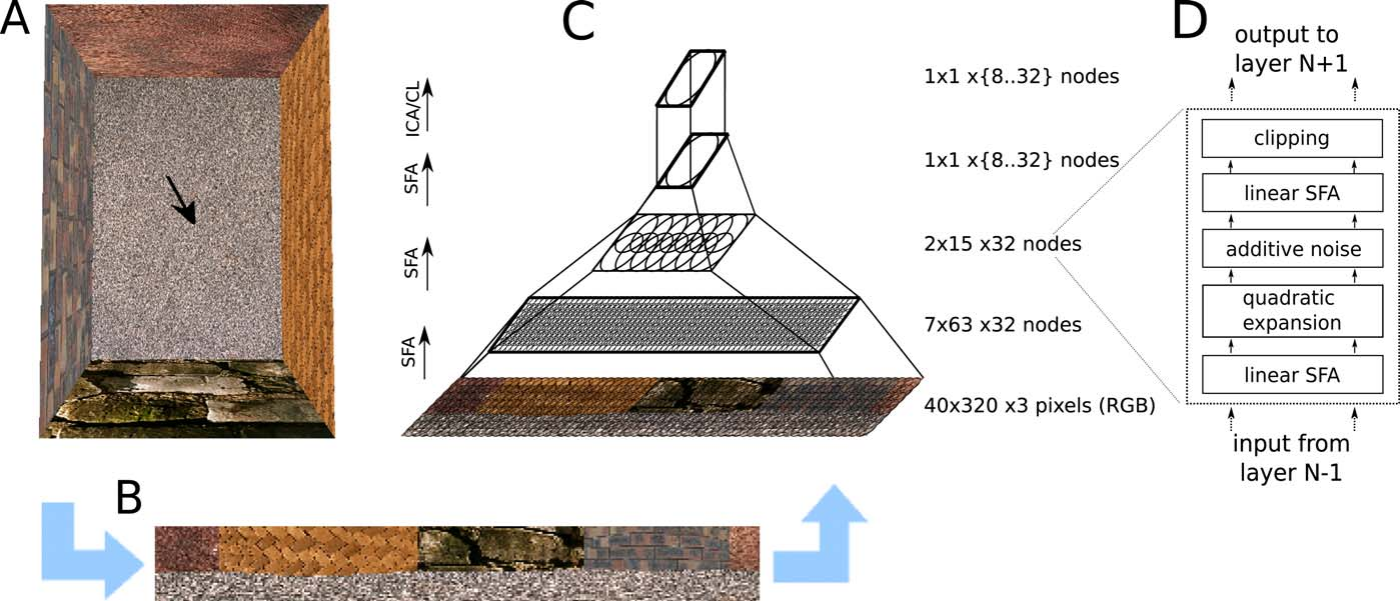
Model Architecture At a given position and orientation of the virtual rat (arrow) in the naturally textured virtual-reality environment (A), input views are generated (B), and processed in a hierarchical network (C). The lower three layers perform the same sequence (D) of linear SFA (for dimensionality reduction), expansion, additive noise, linear SFA (for feature extraction), and clipping; the last layer performs sparse coding (either ICA or CL).

The outcome of an unsupervised learning rule, such as SFA, is crucially determined by the statistics of the training data. As we want to show that oriospatial cells can be learned from raw sensory stimuli, we approximate the retinal stimuli of a rat by video sequences generated in a virtual-reality environment. The input statistics of the training data are thus jointly determined by the structure of the virtual-reality environment and the movement pattern of the simulated rat. As this video data is very high-dimensional, nonlinear SFA in a single step is computationally unfeasible. To overcome this problem, the model is organized as a hierarchy of SFA nodes in analogy to the hierarchy of the brain's visual system (see Figure 2C).

#### Simulated environments.

Many experimental place field data were recorded either in a linear track or in an open field apparatus. For our simulations, we use a linear track of 10:1 side length and a rectangular open field of 3:2 side length. We have also simulated radial mazes (e.g., plus or eight-arm mazes) as a third apparatus type, but they can be considered as a combination of an open field in the center with linear tracks extending from it, and simulation results for this type will not be presented here.

The input data consists of pixel images generated by a virtual-reality system based on OpenGL with textures from the Vision Texture Database [24]. The virtual rat's horizontal FOV is 320° and is consistent with that of a real rat [25] (see Figure 2A for a top view of the environment, and Figure 2B for a typical rat's view from this environment). The vertical FOV is reduced to 40° because outside this range usually only unstructured floor and ceiling are visible. An input picture has 40 by 320 color pixels (RGB, 1 pixel/°). The input dimensionality for the system is thus 38,400, while the dimensionality of the interesting oriospatial parameter space is only three-dimensional (*x*- and *y*-position and orientation).

#### Movement patterns of the virtual rat.

As an approximation of a rat's trajectory during exploration in place-field experiments, we use Brownian motion on the three-dimensional parameter space of position and orientation (i.e., head direction). The virtual rat's position pos(t) at each time step t is updated by a weighted sum of the current velocity and Gaussian white noise noise with standard deviation vr. The momentum term m can assume values between zero (massless particle) and one (infinitely heavy particle), so that higher values of m lead to smoother trajectories and a more homogeneous sampling of the apparatus in limited time. When the virtual rat would traverse the apparatus boundaries, the current velocity is halved and an alternative random velocity update is generated, until a new valid position is reached:

currentVelocity = pos(t) − pos(t−1);

repeat

noise = GaussianWhiteNoise2d() * vr;

pos(t+1) = pos(t) + m * currentVelocity + (1−m) * noise;

if not isInsideApparatus(pos(t+1)):

currentVelocity = currentVelocity / 2;

until isInsideApparatus(pos(t+1))

We call the standard deviation (normalized by room size *L*) of the noise term *translational speed v* **_r_**. In the *simple movement* paradigm, the head direction is calculated analogously (but without checks for traversal of boundaries), and we call the standard deviation of the noise term (in units of 2π) for the head direction trajectory *rotational speed* *v*_ϕ_. On long timescales and with finite room size, this type of movement approximates homogeneous position and orientation probability densities, except at the apparatus boundaries where a high momentum term can increase the position probability. We call the ratio of rotational to translational speed *v_ϕ_*/*v* **_r_** the *relative rotational speed v_rel_*. The actual choice of *v_rel_* is based on the rat's behavior in different environments and behavioral tasks. In linear track experiments, the rat's movement is essentially one-dimensional and the animal rarely turns on mid-track but instead mostly at the track ends. Accordingly, we use a large momentum term, so that the virtual rat often translates smoothly between track ends and rarely turns on mid-track. In the open field, on the other hand, full two-dimensional movement and rotation is possible, but the actual statistics depend on the behavioral task at hand. We mimic the common pellet-chasing experiment [11] by using isotropic two-dimensional translational speed and setting *v_rel_* to a relatively high value.

In the *simple movement* paradigm, head direction and body movement are completely independent, so that head direction can be modeled with unrestricted Brownian motion. We also consider a *restricted head movement* paradigm, in which the head direction is enforced to be within ±90° of the direction of body movement:

currentAngularVelocity = phi(t) - phi(t-1);

repeat

noise = GaussianWhiteNoise1d() * vphi;

phi(t+1) = phi(t) + m * currentAngularVelocity +      (1-m) * noise;

until headDirIsWithin+/-90DegOfMovementDir     (pos(t+1)- pos(t), phi(t+1))

This constraint implicitly restricts the range of possible relative speeds. While it is still possible to have arbitrarily high relative rotational speed by turning often or quickly, very low relative rotational speed cannot be achieved anymore in finite rooms. Typically, if the rat reaches a wall, it has to turn, resulting in a lower bound for the relative rotational speed *v*_*rel*_. To generate input sequences with lower *v*_*rel*_, one needs to discard periods with dominant rotations from the input sequence. For a biological implementation of such a mechanism, the rat's limbic system could access the vestibular rotational acceleration signal in order to downregulate the learning rate during quick turns. We will refer to this mechanism as *learning rate adaptation* (LRA).

A third movement statistics can be generated if we assume that an animal looks at objects or locations in the room for some time while moving around. During this period the animal fixates a specific location *X* in the room, i.e., it always turns its head into the direction of *X*, independently of its position. We implement *X* as a fixation point on the wall that moves in the following way: first, we generate an orientation *ϕ* using the algorithm and the same parameters as for the head-direction cell simulations. Second, the point *X* is defined as the point on the wall the rat would fixate if it were in the center of the room with head direction *ϕ*. We employ the identical translational movement mechanism as above, whereas the head direction is now completely determined by the animal position and the position of the viewpoint *X*. In this paradigm, both position and orientation are dependent and vary rather quickly, while the position of *X* changes slowly. We call this movement pattern *spatial view* paradigm and suggest that it is a more appropriate description of a primate's movement pattern than the previous two.

#### Model architecture and training.

Our computational model consists of a converging hierarchy of layers of SFA nodes and a single final sparse coding step (see Figure 2C). Each SFA node finds the slowest output features from its input according to the SFA algorithm given in the section Slow Feature Analysis and performs the following sequence of operations: linear SFA for dimensionality reduction, quadratic expansion with subsequent additive Gaussian white noise (with a variance of 0.05), another linear SFA step for slow-feature extraction, and clipping of extreme values at ±4 (see Figure 2D). Effectively, a node implements a subset of full quadratic SFA. The clipping removes extreme values that can occur on test data very different from training data.

In the following, the part of the input image that influences a node's output will be denoted as its *receptive field*. On the lowest layer, the receptive field of each node consists of an image patch of 10 by 10 pixels with three color dimensions each. The nodes form a regular (i.e., non-foveated) 7 by 63 grid with partially overlapping receptive fields that jointly cover the input image of 40 by 320 pixels. The second layer contains 2 by 15 nodes, each receiving input from 3 by 8 layer 1 nodes with neighboring receptive fields, resembling a retinotopical layout. All layer 2 output converges onto a single node in layer 3, whose output we call *SFA-output*. Thus, the hierarchical organization of the model captures two important aspects of cortical visual processing: increasing receptive field sizes and accumulating computational power at higher layers.

The network's SFA-output is subsequently fed into a final computational node that performs linear sparse coding, either by applying Independent Component Analysis (ICA) (we use CuBICA which is based on the diagonalization of third and fourth order cumulants [26]) or by performing competitive learning (CL). The top-layer output will be called *ICA-output* or *CL-output*, respectively. ICA applied to nonlocalized grid-cell inputs finds sparser codes than CL, but the latter seems biologically more realistic. More details on different approaches for sparse coding of grid-cell input can be found in [27].

The layers are trained sequentially from bottom to top on different trajectories through one of the simulated environments. For computational efficiency, we train only one node with stimuli from all node locations in its layer and replicate this node throughout the layer. This mechanism effectively implements a weight-sharing constraint. However, the system performance does not critically depend on this mechanism. To the contrary, individually learned nodes *improve* the overall performance.

In analogy to a rat's brain, the lower two layers are trained only once and are kept fixed for all simulations presented here (like the visual system, which remains rather stable for adult animals). Only the top SFA and ICA layer are retrained for different movement statistics and environments. For our simulations, we use 100,000 time points for the training of each layer. Since training time of the entire model on a single PC is on the order of multiple days, the implementation is parallelized and training times thus reduced to hours. The simulated rat's views are generated from its configuration (position and orientation) with floating point precision and are not artificially discretized to a smaller configuration set.

The network is implemented in Python using the MDP toolbox [28], and the code is available upon request.

#### Analysis methods.

The highly nonlinear functions learned by the hierarchical model can be characterized by their outputs on the three-dimensional configuration space of position and head direction. We will call two-dimensional sections of the output with constant (or averaged) head direction *spatial firing maps* and one-dimensional sections of the output with constant (or averaged) position *orientation tuning curves*. For the sparse coding results with ICA, the otherwise arbitrary signs are chosen such that the largest absolute response is positive.

The sensitivity of a function *f* to spatial position **r** will be characterized by its mean positional variance *η* **_r_**, which is the variance of *f*(**r**,*ϕ*) with respect to **r** averaged over all head directions *ϕ*: *η* **_r_**(*f*) = 〈var**_r_** (*f*(**r**,*ϕ*))〉*_ϕ_*. Correspondingly, the sensitivity of a function *f* to head direction *ϕ* will be characterized by its directional variance *η_ϕ_* averaged over all spatial positions **r**: *η_ϕ_*(*f*) = 〈var*_ϕ_* (*f*(**r**,*ϕ*))〉**_r_**. A perfect head-direction cell has no spatial structure and thus a vanishing *η* **_r_** and positive *η_ϕ_*, while a perfect place cell has positive *η* **_r_** due to its spatial structure but no orientation dependence and thus a vanishing *η_ϕ_*.

### Theoretical Methods

Considering the complexity of the computational model presented in the last section, one might expect that it would be impossible to make any analytical statement about the model's behavior. However, in this section we introduce a mathematical framework that actually allows us to make detailed predictions depending on the movement statistics of the simulated rat. The theoretically less inclined reader should feel free to skip all sections marked by a * without loss of the general understanding of our model and the results.

#### The modified optimization problem*.

Consider a rat in an environment that is kept unchanged for the duration of the experiment. The visual input the rat perceives during the experiment is the input signal for the learning task stated above. This section addresses the following question: can we predict the functions learnt in such an experiment, and, in particular, will they encode the rat's position in a structured way?

As the rat's environment remains unchanged for the duration of the experiment, the visual input cannot cover the full range of natural images but only the relatively small subset that can be realized in our setup. Given the environment, the rat's visual input can at all times be uniquely characterized by the rat's position and its head direction. We combine these parameters in a single *configuration vector* **s** and denote the image the rat perceives when it is in a particular configuration **s** as **x**(**s**). We refer to the manifold of possible configurations as *configuration space V*. Note that *V* in general does not have the structure of a vector space.

In a sufficiently complex environment, we can not only infer the image from the configuration but also the configuration from the image, so that there is a one-to-one correspondence between the configurations and the images. If we are not interested in how the functions the system learns respond to images other than those possible in the experiment, we can think of them as functions of the configuration **s**, since for any function 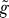 (**x**) of the images, we can immediately define an equivalent function *g*(**s**) of the configuration:

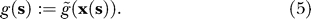



This leads to a simplified version of our problem. Instead of using the images **x**(*t*), we use the configuration **s**(*t*) as an input signal for our learning task.

It is intuitively clear that functions that vary slowly with respect to the configuration **s** will create slowly varying output when applied to **s**(*t*) as an input signal, because **s**(*t*) is continuous in time. Mathematically, this is reflected by the chain rule:

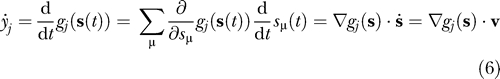
where ∇*g_j_* is the gradient of *g_j_* and 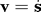 is the velocity in configuration space (note the difference in notation to ∇· **A**(**s**), which denotes the divergence of a vector-valued function **A**).

In order to generate slowly varying output, *g_j_* should vary slowly with **s** in configuration regions with large velocities **v** and reserve stronger gradients for regions with small velocities. Thus, the optimal functions depend on the velocity statistics of the input signal. As their dependence on the detailed time-course of the input signal **s**(*t*) is inconvenient to handle mathematically, we assume that the duration of the experiment is long enough to do statistics on the behavior of the rat. Its motion can then be described by means of a joint probability density function *p*_**s,v**_(**s**,**v**), which quantifies how often the rat is found in a particular configuration **s** and moves with velocity **v**. If the movement of the rat is ergodic, we may equivalently replace the temporal averages in the original formulation of the learning task by weighted averages over all configurations and velocities:

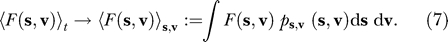



If we take the average of a function that does not explicitly depend on the velocity **v** (i.e., *F*(**s**,**v**) = *F*(**s**)), we can simplify the average 〈*F*(**s**)〉**_s_**_,**v**_ by integrating over the velocity:

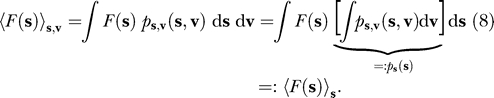
Here *p* **_s_** is the marginal probability of finding the rat in configuration **s**, irrespective of its velocity.

Making use of Equations 5–8, we can now state an equivalent alternative formulation of the learning task.

***Optimization problem 2.*** *Given a function space* ℱ *on a configuration space V, which is sampled with probability density p* **_s_**_,**v**_(**s**,**v**), *find a set of J functions g_j_*(**s**) ∈ ℱ *that*

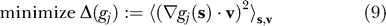

*under the constraints*

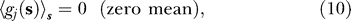


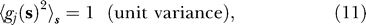


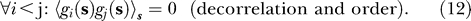



If we do not impose any restriction on the function space ℱ (apart from sufficient differentiability and integrability), this modified optimization problem can be solved analytically for a number of cases. Following a previous analytical treatment [29], we refer to the optimal functions in the unrestricted function space as Δ-*optimal functions*; they are shown in the Results section together with the numerical simulations.

#### A differential equation for the optimal functions*.

In this section we apply variational calculus to optimization problem 2 and derive a partial differential equation for the optimal functions *g_j_*. We prove that the optimization problem can be simplified to an eigenvalue problem of a partial differential operator 𝒟 whose eigenfunctions and eigenvalues form the Δ-optimal functions and their Δ-values, respectively. For the sake of brevity, we shift the proofs to Protocol S1, so that the reader can focus on the main theorems.

Using Lagrange multipliers we get an objective function for the functions *g_j_* that incorporates the constraints:

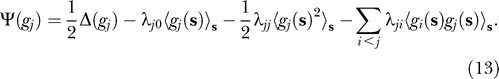
Here, factors 1/2 have been introduced for mathematical convenience and have no influence on the results.

In the following, we will not need the full dependence of the probability density *p* **_s_**_,**v**_ on the velocity, but only the following function:

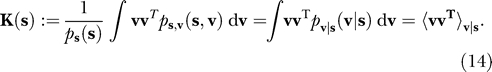

**K** is the matrix containing the second-order moments of the conditional velocity distribution *P*(**v**|**s**) = *P*(**s**,**v**)/*P*(**s**). It contains information on how fast and in which direction the rat typically moves given it is in configuration **s**.

Applying variational calculus to the objective function of Equation 13, we can derive a necessary condition for the solutions of optimization problem 2.

***Theorem 1.*** *For a particular choice of the parameters λ_ij_, the solutions g_j_ of optimization problem 2 obey the Euler-Lagrange equation*

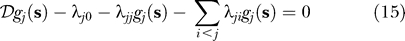

*with the boundary condition*

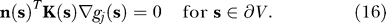

*Here, the partial differential operator* 𝒟 *is defined as*

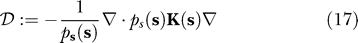

*and* **n**(**s**) *is the unit normal vector on the boundary* ∂*V of the configuration space V*.

We now show that the solutions of optimization problem 2 are given by the eigenfunctions of the operator 𝒟. The essential observation we need is stated in Theorem 2.

***Theorem 2.*** *Let* ℱ*_b_* ⊂ ℱ *be the space of functions that obey the boundary condition Equation 16. Then* 𝒟 *is self-adjoint on* ℱ*_b_ with respect to the scalar product*

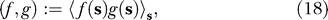

*i.e.,*

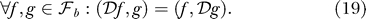



This property is useful, as it allows the application of the spectral theorem known from functional analysis, which states that any self-adjoint operator possesses a complete set of eigenfunctions *f_j_*(**s**) ∈ ℱ*_b_* with real eigenvalues Δ*_j_*, which are pairwise orthogonal, i.e., a set of functions that fulfills the following conditions:

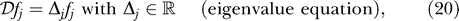


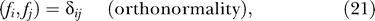


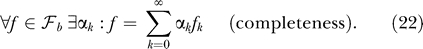



Because the weighted average over configurations is equivalent to a temporal average, the scalar product (Equation 18) is essentially the covariance of the output of the functions *f* and *g* (if they have zero mean). The orthonormality (Equation 21) of the eigenfunctions thus implies that the eigenfunctions fulfill the unit variance and decorrelation constraint. This is stated in Theorem 3.

***Theorem 3.*** *Apart from the constant function, which is always an eigenfunction, the (adequately normalized) eigenfunctions f_j_* ∈ *ℱ of the operator 𝒟 fulfill the constraints of Equations 10–12.*

If we set *λ_j_*_0_ = *λ_ji_* = 0 for *i* ≠ *j*, the eigenfunctions also solve Equation 15, making them good candidates for the solution of optimization problem 2. To show that they indeed minimize the Δ-value, we need Theorem 4.

***Theorem 4.*** *The* Δ-*value of the normalized eigenfunctions f_j_ is given by their eigenvalue* Δ*_j_*.

At this point, it is intuitively clear that the eigenfunctions with the smallest eigenvalues form the solution to optimization problem 2. This is stated in Theorem 5.

***Theorem 5.*** *The J eigenfunctions with the smallest eigenvalues* Δ*_j_* ≠ 0 *are a solution of optimization problem 2.*

The advantage of this approach is that it transfers the original optimization problem to that of finding the eigenfunctions of a partial differential operator. This type of problem is encountered frequently in other contexts and has been studied extensively.

It is worth noting that the formalism described here is not restricted to the example used here. As it is independent of the concrete nature of the configuration space, it can be applied to more complicated problems, e.g., to a rat moving in an environment with moving objects, whose positions would then be additional components of the configuration **s**.

#### Qualitative behavior of the solutions for inhomogeneous movement statistics*.

Structurally, Equation 20 is a wave equation that describes the eigenmodes of an inhomogeneous membrane, which generally show oscillatory behavior. A brief calculation for a one-dimensional configuration space with *p*_s_ and **K** independent of **s** shows that the wavelength of the oscillation is given by 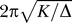 . It is reasonable to assume that this behavior will be preserved qualitatively if *p*_s_ and **K** are no longer homogeneous but depend weakly on the configuration. In particular, if the wavelength of the oscillation is much shorter than the typical scale on which *p*_s_ and **K** vary, it can be expected that the oscillation “does not notice” the change. Of course, we are not principally interested in quickly varying functions, but they can provide insights into the effect of variations in *p*_**s**_ and **K**.

To examine this further, we consider the eigenvalue Equation 20 for a one-dimensional configuration space and multiply it by *p*_**s**_:

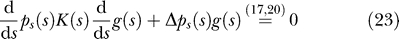



We can derive an approximate solution of this equation by treating 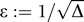 as a small but finite perturbation parameter. This corresponds to large Δ-values, i.e., quickly varying functions. For this case we can apply a perturbation theoretical approach that follows the scheme of the Wentzel-Kramers-Brillouin (WKB) approximation used in quantum mechanics. Knowing that the solution shows oscillations, we start with the complex ansatz

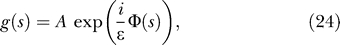
where Φ(*s*) is a complex function that needs to be determined.

Treating *ɛ* as a small number, we can expand Φ in orders of *ɛ*

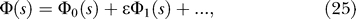
where again the ellipses stand for higher-order terms. We insert this expansion into Equation 23 and collect terms of the same order in *ɛ*. Requiring each order to vanish separately and neglecting orders *ɛ* ^2^ and higher, we get equations for Φ_0_ and Φ_1_:

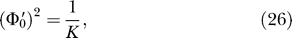


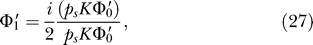
where the prime denotes the derivative with respect to *s*. These equations are solved by

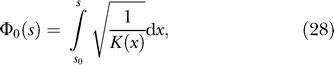


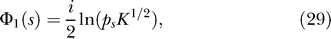
where *s*_0_ is an arbitrary reference point. Inserting this back into Equation 24, we get the approximate solution

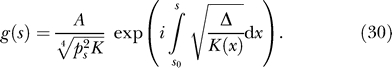



This shows that the solutions with large Δ-values show oscillations with local frequency 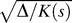 and amplitude 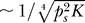 . As large values of *K* indicate that the rat moves quickly, this implies that the local frequency of the solutions is smaller in regions with larger velocities whereas small velocities, e.g., close to walls, lead to higher frequencies than expected for homogeneous movement. Intuitively, this means that the functions compensate for quick movements with smaller spatial frequencies such that the effective temporal frequency of the output signal is kept constant.

Understanding the dependence of the amplitude on *p_s_* and *K* is more subtle. Under the assumption that *K* is independent of *s*, the amplitude decreases where *p_s_* is large and increases where *p_s_* is small. Intuitively, this can be interpreted as an equalization of the fraction of the total variance that falls into a small interval of length 
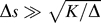
 . This fraction is roughly given by the product of the probability *p*(*s*)Δ*s* of being in this section times the squared amplitude

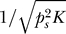
of the oscillation. For constant *K*, this fraction is also constant, so the amplitude is effectively rescaled to yield the same “local variance” everywhere. If *p_s_* is constant but *K* varies, on the other hand, the amplitude of the oscillation is small in places where the rat moves quickly and large where the rat moves slowly. This corresponds to the intuition that from the perspective of slowness there are two ways of treating places where the rat moves quickly: decreasing the spatial frequency to generate slower output signals and/or decreasing the amplitude to “pay less attention” to these regions. There is also a strong formal argument why the amplitude should depend on 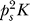 . As the optimization problem is invariant under arbitrary invertible nonlinear coordinate changes, the amplitude of the oscillation should depend only on a function of *p_s_* and *K* that is independent of the coordinate system. This constrains the amplitude to depend on 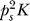 , as this is the only combination that is invariant under coordinate changes.

The key insight of this analysis is that the optimal functions show oscillations that are spatially compressed in regions where the rat moves with low velocities. This implies that the spatial resolution of the SFA solutions is higher in those regions. Consequently, the size of the place fields after sparse coding should be smaller in regions with small velocities, which might explain smaller place fields near arena boundaries [8,30]. If we assume the animal moves faster parallel to a wall of the arena than perpendicular to it, our theory predicts elongated place fields along the walls that might be similar to the crescent-shaped fields reported in [31] for a circular arena.

## Results

We apply our theoretical framework and computer simulations to a number of environments and movement patterns that resemble typical place-cell experiments. In the next section, Open Field, we show results for the open field, beginning with the mathematical analysis and simulation results for the simple movement paradigms with high and low relative speeds. Subsequently, the simulation results for the restricted head movement paradigm, including LRA, and the spatial view paradigm are shown. In the section Linear Track, the results for the linear track with its two-dimensional configuration space are shown.

### Open Field

One of the most common environments for place-cell experiments is an open-field apparatus of rectangular or circular shape. Here, the most typical experimental paradigm is to throw food pellets randomly into the apparatus at regular intervals, leading to a random search behavior of the rat. For this case, the rat's oriospatial configuration space comprises the full three-dimensional manifold of position and orientation. In this section, we present results from experiments with simulated rat trajectories at either high or low relative rotational speeds leading to undirected place cells or position invariant head-direction cell-type results, respectively.

#### Theoretical predictions for the simple movement paradigm*.

In a rectangular open field, the configuration space can be parametrized by the animal's position, indicated by the coordinates *x* and *y*, and by its head direction *ϕ*. The total configuration space is then given by **s** = (*x*,*y*,*ϕ*) ∈ [0,*L_x_*] × [0,*L_y_*] × [0,2π[. *L_x_* and *L_y_* denote the size of the room in *x*- and *y*-direction, respectively. We choose the origin of the head direction *ϕ* such that 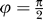 corresponds to the rat looking to the North. The velocity vector is given by **v** 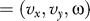 , where *v_x_*, *v_y_* denote the translation velocities and *ω* is the rotation velocity. For the typical pellet-throwing experiment, we make the approximation that the velocities in the three different directions are decorrelated and that the rat's position and head direction are homogeneously distributed in configuration space. Moreover, in an open field there is no reason why the variance of the velocity should be different in *x*- and *y*-directions. The covariance matrix of the velocities then takes the form

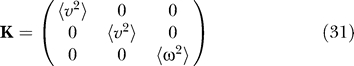
and the probability density *p*(*x*,*y*,*ϕ*) is a constant.

In this case, the eigenvalue problem (Equation 20) for the operator 𝒟 takes the following form:

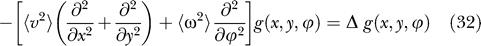
with the boundary conditions (Equation 16) yielding

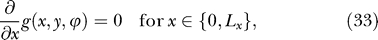


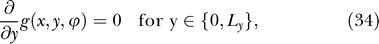
and cyclic boundary conditions in the angular direction.

It is easy to check that the eigenfunctions and the corresponding Δ-values are given by

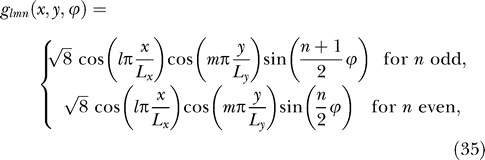


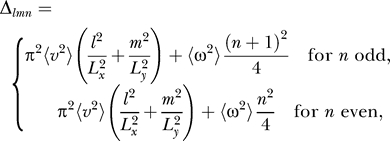
with *l*, *m*, and *n* being nonnegative natural numbers. Only *l* = *m* = *n* = 0 is not allowed, as this case corresponds to the constant solution, which violates the unit variance constraint.

To predict the actual outcome of the simulations, we need to order these solutions by their Δ-values. For better comparability with the simulation results, it is convenient to rewrite the Δ-values in the following form:

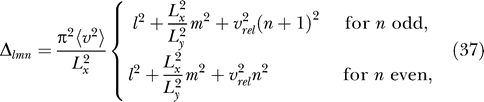
where

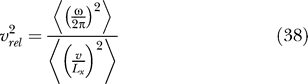
denotes the relative rotational speed, i.e., the ratio of the root mean square of rotational and translational velocity, if translational velocity is measured in units of the room size in *x*-direction per second and rotational velocity is measured in full circles per second.

**Figure 3:**
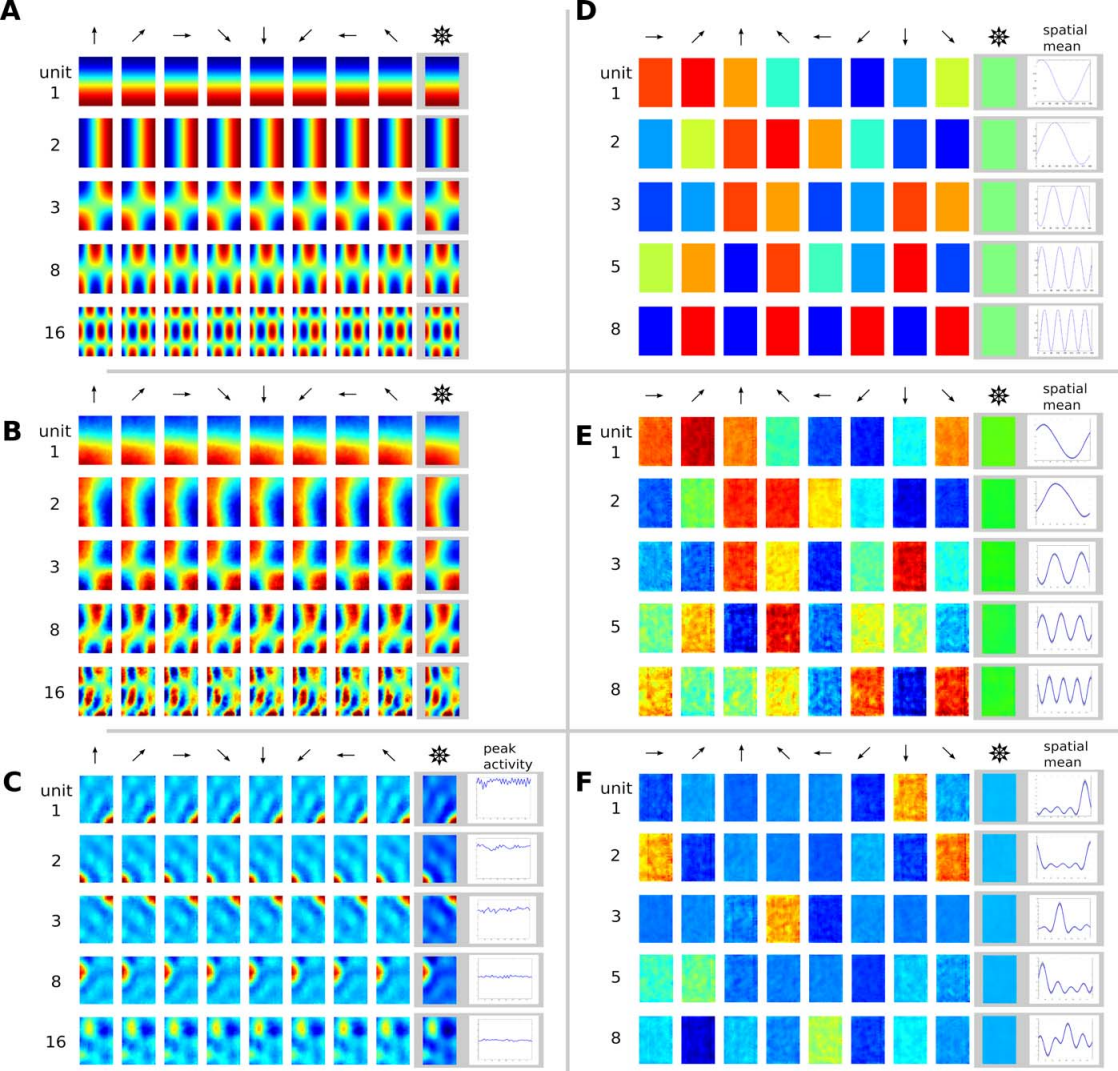
Theoretical Predictions and Simulation Results for the Open Field with the Simple Movement Paradigm (Independent Translation and Head Direction), Separately Learned Place Cells and Head-Direction Cells, and ICA for Sparsification Each row within each panel shows the response of one unit as a function of position for different head directions (indicated by arrows), as well as the mean value averaged over all head directions (indicated by the superimposed arrows). Blue denotes low activity, green intermediate activity, and red high activity. (C) also shows orientation tuning curves at the position of a unit's maximal activity. (D–F) also show orientation tuning curves averaged over all positions ± one standard deviation. (A) Theoretical prediction for the SFA layer with relatively quick rotational speed compared with translational speed (*v* *_rel_* = 32). Solutions are ordered by slowness. All solutions are head-direction invariant and have regular rectangular grid structures. (B) Simulation results for the SFA layer for the same settings as in (A), ordered by slowness. The results are similar to the theoretical predictions up to mirroring, sign, and mixing of almost equally slow solutions. All units are head-direction invariant and code for spatial position but are not localized in space. (C) Simulation results for the ICA layer for the same simulation as in (B), ordered by sparseness (kurtosis). Firing patterns of all units are head-direction invariant and localized in space, resembling hippocampal place cells. (D) Theoretical prediction for the SFA layer for relatively slow rotational speed compared with translational speed. Solutions are ordered by slowness. All solutions are position invariant and constitute a Fourier basis in head-direction space. As the phases of the theoretical solutions are not uniquely determined, they were adjusted to match the simulation results in (E). (E) Simulation results for the SFA layer for the same settings as in D (*v* *_rel_* = 0.08), ordered by slowness. The results are similar to the theoretical predictions. All units are position invariant and head-direction specific but not localized in head-direction space, i.e., all units except 1 and 2 have multiple peaks. (F) Simulation results for the ICA layer for the same simulation as in (E) ordered by sparseness (kurtosis). Firing patterns of all units are position invariant and localized in head-direction space resembling subicular head-direction cells.

We can now discuss two limit cases in terms of the relative velocity *v_rel_*. Let us first consider the case where the rat moves at small velocities while making a lot of quick turns, i.e., *v*_*rel*_ ≫ 1. In this case, the smallest Δ-values can be reached by setting *n* = 0 unless 
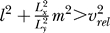
 . Since for *n* = 0 the functions *g_lmn_* do not depend on the angle *ϕ*, the slowest functions for this case are invariant with respect to head direction and lead to place cells, see below. The behavior of the solutions and the respective simulation results are depicted in Figure 3A and 3B.

In the other extreme, *v_rel_* is much smaller than one, i.e., the rat runs relatively fast while making few or slow turns. The smallest Δ-values can then be reached by choosing *l* = *m* = 0 unless *n* ^2^ > 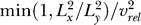 . The corresponding functions are invariant with respect to position while being selective to head direction, a feature that is characteristic for head-direction cells. A comparison of these theoretically predicted functions with simulation results are shown in Figure 3D and 3E.

#### Simulation results for the simple movement paradigm.

It is intuitively clear and has been shown in the last section that for high relative orientational speed *v_rel_* the system output becomes slowest if it is invariant to head direction and only codes for spatial position. For low *v_rel_* on the other hand, invariance for position while coding for head orientation is the best solution to the optimization problem.

In Figure 3B, the spatial firing maps of SFA output units from the simulation with high *v_rel_* = 32 are shown. Here, all units are almost completely orientation invariant and resemble the theoretical predictions from Figure 3A. The first unit has low activity when the simulated rat is in the South of the apparatus, is most active in the North, and shows a gradual increase in the shape of a half cosine wave in between. The unit is invariant to movements in the East–West direction. The second unit behaves similarly, but its activity pattern is rotated by 90°. The following units have more spatial oscillations and somewhat resemble grid cells, which are not localized. Figure 3C shows ICA output units from the same simulation as in Figure 3B. All units are orientation invariant, just as is their input from the first 16 SFA units, but most have only a single peak of activity and each at a different position. The sparser units are more localized in space while less sparse units have larger firing fields or multiple peaks. These results closely resemble place cells from rodent's hippocampal areas CA1 and CA3.

In Figure 3E, SFA output units from the simulation with low relative rotational speed *v_rel_* = 0.08 are shown. In this case, all units are almost completely position invariant but their response oscillates with the orientation of the rat. The first unit changes activity with the sine of orientation and the second unit is modulated like a cosine. Unit 3 has twice the frequency, unit 5 has a frequency of three, and unit 8 has a frequency of four. Again, the simulation results reproduce the theoretical predictions shown in Figure 3D. Figure 3F shows ICA output units from the same simulation as in Figure 3E. All units are position invariant like their inputs from the first eight SFA units, but most have only a single peak of activity and each at a different orientation. The sparser units are more localized in orientation while later ones have broader tuning curves. These results closely resemble head-direction cells from rodent's subicular areas.

#### Simulation results for the restricted head movement paradigm.

In the previous section we used independent head direction and body movement and used different movement statistics for different cell types, such as fast rotational speed for place cells and slow rotational speed for head-direction cells. This allowed us to obtain nearly ideal simulation results that match closely the theoretical predictions, but it is unrealistic for two reasons. First, in a real rat, head-direction and movement direction are correlated. Second, in a real rat, place cells and head-direction cells have to be learned simultaneously and thus with the same movement pattern.

**Figure 4:**
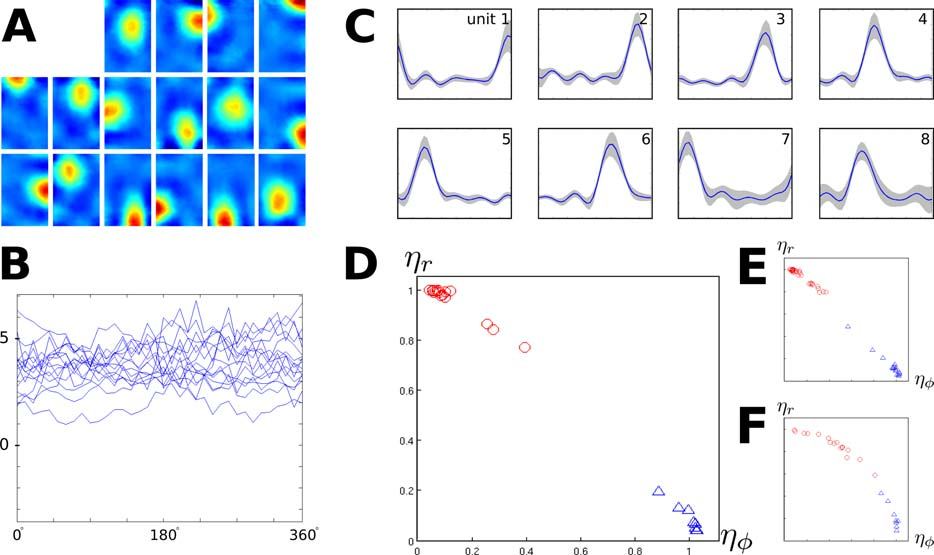
Simulation Results for the Open Field with More Realistic Movement Patterns and Competitive Learning for Sparsification in the Last Layer The network was trained with a movement pattern of relatively high rotational speed. Two distinct populations of cells were trained, one as before, the other was trained with LRA in the top SFA layer, reducing the impact of periods with high rotational speed. (A) Simulation results for the top layer CL units without LRA. Each subplot shows the mean spatial firing rate of one output unit averaged over all orientations. The slowest 16 SFA outputs were used for CL, and 16 CL units were trained. All units are localized in space, closely resembling hippocampal place cells. Blue color denotes low activity, green intermediate activity, and red high activity. (B) Orientation tuning of the units shown in (A). Firing patterns of all units are mostly head direction invariant. (C) Simulation results for the top layer CL units with LRA in the top SFA layer. Each subplot shows the mean orientation tuning curve in blue, and a gray area indicates ± one standard deviation. The slowest eight SFA outputs were used for CL, and eight CL units were trained. Firing patterns of all units are mostly position invariant and localized in head-direction space closely resembling subicular head-direction cells. (D) Scatterplot of mean directional variance *η_ϕ_* and mean positional variance *η*_r_ (see Methods) of the results shown in (A) (red circles) and (C) (blue triangles). Units from (A) cluster in an area with high positional variance *η*_r_ and low orientational variance *η_ϕ_*, while units from (C) cluster in an area with low positional variance *η*_r_ and high orientational variance *η_ϕ_*. (E) Scatterplot of *η_ϕ_* and *η*_r_ for the same simulation parameters as in (A–D) but with more CL output units. 32 units were trained without LRA (red circles) and 16 with LRA (blue triangles). The solutions lie in similar areas as in (D). (F) Scatterplot of *η_ϕ_* and *η*_r_ for the same simulation parameters as in (A–D), but with more SFA outputs used for CL. 32 SFA units were used without LRA (red circles) and 16 with LRA (blue triangles). The solutions show mixed dependence on position and head direction but are still clearly divided into a mostly head-direction invariant population (red) and a mostly position invariant population (blue).

In this section we introduce three changes for more realism. First, a more realistic movement pattern is used, where the rat's head is enforced to be within 90° of the current body movement (see Methods) and the relative rotational speed *v_rel_* is set to an intermediate value of 0.6. Second, place cells and head-direction cells are learned on the same input statistics and LRA is used in the top SFA layer for the head-direction cell population (see Methods). Third, ICA for sparse coding in the last layer is replaced by CL. Simulation results are shown in Figure 4.

As the relative rotational speed *v_rel_* is smaller than in the previous section, some SFA solutions (unpublished data) change with head direction: unit 16 of 32 is the first unit with noticeable head-direction dependence, while none of the first 32 SFA solutions in the place-cell simulation in the last section was head-direction dependent. In Figure 4A, the spatial firing maps for all units trained without LRA are shown averaged over all orientations. The corresponding orientation tuning curves (measured at the peak of the place field) are given in Figure 4B. All units are localized in space and are largely independent of orientation with activity centers distributed evenly in the room.

Figure 4C shows the simulation results with identical movement statistics but with LRA turned on in the top SFA layer, so that learning is downregulated at timepoints with rapid head-direction changes. Tuning curves of all units are shown together with the spatial standard deviation of activity, which is generally very small. All units are localized in head-direction space and mostly position independent, with approximately even spacing of directions of maximum activity. The LRA can eliminate the effect of head rotation only to some extent and thus SFA units 7 and 8 (unpublished data) show significant dependence on position, while the slowest unit affected by position in the previous section was unit 15.

A scatterplot of the mean positional variance *η*_r_ versus mean orientational variance *η* *_ϕ_* (see Methods) of the units from Figure 4A and Figure 4C is shown in Figure 4D. Perfect head-direction cells would be located in the bottom right, while perfect place cells would be located in the top left. Red circles denote the simulated place cells from Figure 4A. Blue triangles denote the simulated head-direction cells from Figure 4C. Both populations cluster near the positions of optimal solutions in the corners.

How does the number of inputs to the last layer (i.e., the number of SFA outputs used) and the number of CL outputs influence the results? Figure 4E shows the same analysis for a simulation with identical settings except the number of CL output units was doubled to 32 without LRA and 16 with LRA, respectively. Most units lie in a similar area as in Figure 4D, but the clusters are denser, since the number of units has doubled. In Figure 4F, the number of output units is again the same as in Figure 4D, but the number of SFA outputs for the last layer is doubled to 32 for the simulation without LRA and 16 for the simulation with LRA. The output units now get inputs from higher, i.e., quicker, SFA units, which tend to depend on both position and orientation. As a result, the CL units span the entire spectrum of completely position invariant to completely orientation invariant solutions, with the more position dependent solutions coming from the simulations without LRA, and the more head-direction dependent solutions coming from the LRA simulation. We have no conclusive explanation, though, why the shape of the data distribution seemingly changes from linear (Figure 4D and 4E) to convex (Figure 4F) with increasing numbers of SFA units. We conclude that the number of CL output units mostly determines the density of place cells but not the qualitative behavior of the solutions, while the number of SFA outputs directly affects the invariance properties of the solutions.

#### Simulation results for the spatial view paradigm.

The previous sections have shown that the same learning mechanism in the same environment, but with different movement statistics, results in either head-direction or place cell–like representations. Although the last section introduced certain restrictions on the head direction, body position and head direction remained mostly independent.

In the following simulation, the virtual animal fixates a location *X* on a wall while it moves through the room. The position of *X* is subject to a random walk on the wall with the same statistics as the head direction in the simple movement paradigm with small *v_rel_* (see Methods). The animal's position is also changed with the same statistics as position in the simple movement paradigm, and the actual head direction is thus determined by the current position and currently fixated point *X*.

**Figure 5:**
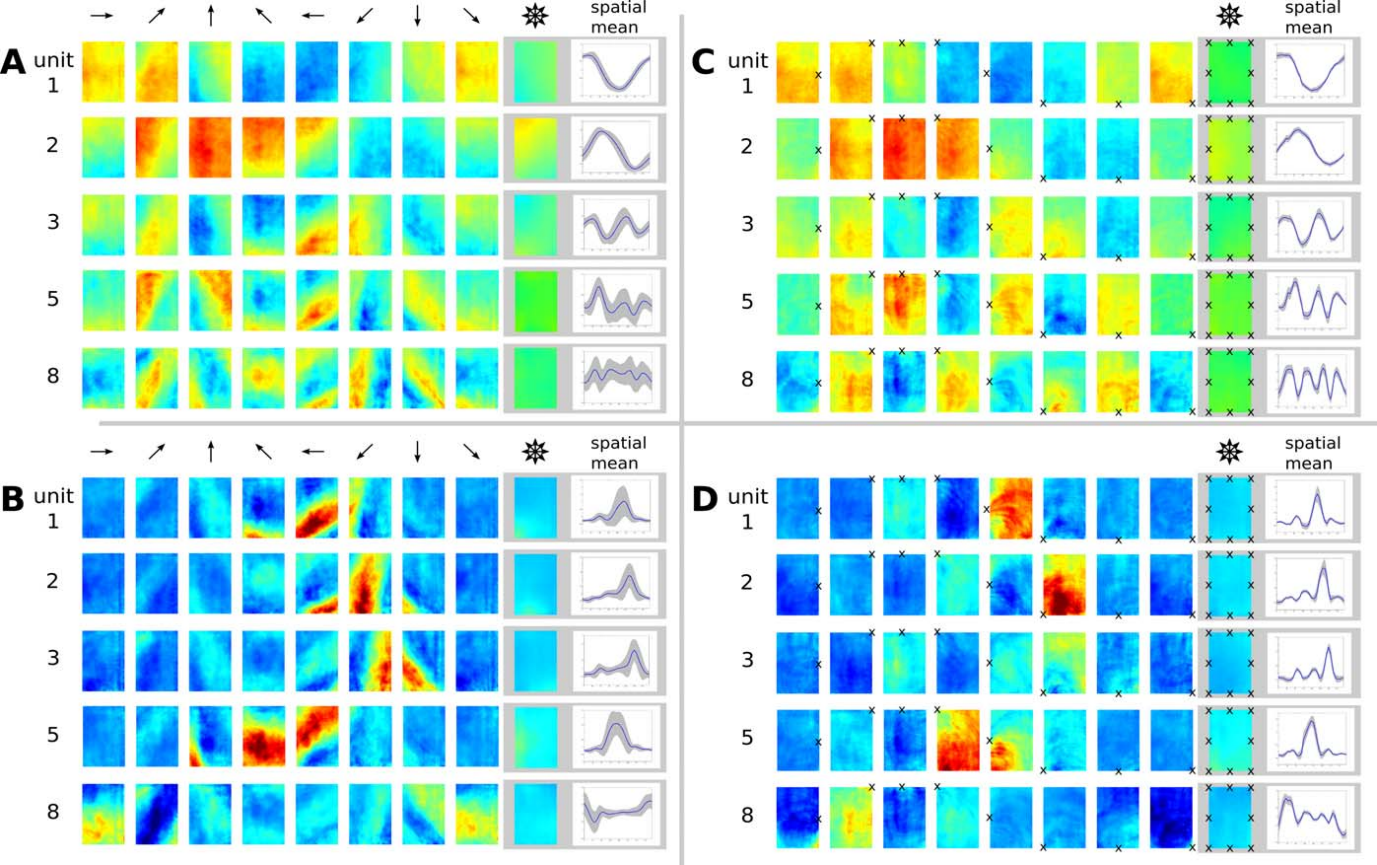
Simulation Results for the Open Field with Trajectories Where Spots on the Wall Were Fixated Blue color denotes low activity, green intermediate activity, and red high activity. (A) Spatial firing map of five representative SFA output units for different “global head directions” (indicated by arrows) and averages over orientations and space. No unit shows spatial or orientation invariance when plotting position and “global head direction” as in previous Figures. (B) ICA results plotted with “global head direction.” (C) Same results as in (A) but plotted with “local head direction” (at each position oriented toward fixation point ×). (D) Same results as in (B) but using the plot method from (C). All units code for a specific view closely resembling primate spatial-view cells.

Note that the configuration space consisting of position and viewpoint has the same structure as the one consisting of position and head direction for the simple movement paradigm. Accordingly, the theoretical predictions for the two scenarios are identical if head direction is “replaced'” by the fixation point. In Figure 5C, we plot the spatial activity pattern such that at each position the rat fixates a specific location marked by an ×. As expected, these plots are virtually identical to the head direction cell plots in Figure 3D and 3E in that activity is largely invariant to position. This can also be seen by the corresponding tuning curves that show small standard deviations (indicated by gray areas). However, while in Figure 3D and 3E the activities are modulated by head direction, activities in Figure 5C depend on the position of viewpoint. If we plot the same data with fixed head direction instead of fixed viewpoint, (Figure 5A), the structure of the activity patterns is obscured. Units 3–5 in Figure 5A, for example, show clear diagonal stripes and correspondingly larger standard deviations in their tuning curves. These SFA solutions jointly code for “view space,” but as before the SFA results are not localized.

Figure 5B and 5D show the results of the ICA layer. The “global direction” plot in Figure 5B is as inadequate as in Figure 5A, while the plot in Figure 5D clearly illustrates the behavior of these cells. Unit 2, for example, is active only when the rat looks at the bottom left corner of the rectangular room, independent of the animal's position. This cell type resembles spatial-view cells found in the primate hippocampal formation (e.g., [32]).

### Linear Track

In a linear track, the rat's movement is essentially restricted to two degrees of freedom, a spatial one and an orientational one. In experimental measurements, the orientational dimension is often collapsed into a binary variable indicating only the direction of movement. In the linear track, these two dimensions are thus experimentally much easier to sample smoothly than the full three-dimensional parameter space of the open field.

#### Theoretical predictions for the linear track*.

In principle, the configuration space for the linear track is the same as for the open field, only with a small side length *L_x_* in one direction. Equation 36 shows that for small *L_x_* the solutions that are not constant in the *x*-direction, i.e., the solutions with *l* ≠ 0, have large Δ-values and thus vary quickly. Therefore, slow functions will be independent of *x,* and we will neglect this dimension and restrict the configuration space to position in *y*-direction and head direction *ϕ*.

Another difference between the simulation setup for the open field and the linear track lies in the movement statistics of the rat. Due to the momentum of the Brownian motion, the rat rarely turns on mid-track. In combination with the coupling between head direction and body motion, this implies that given the sign of the velocity in *y*-direction the head direction is restricted to angles between either 0 and *π* (positive velocity in *y*-direction, North) or between *π* and 2*π* (negative velocity in *y*-direction, South). If, in addition, the rat makes a lot of quick head rotations, the resulting functions can only be slowly varying if they are invariant with respect to head direction within these ranges. This leaves us with a reduced configuration space that contains the position *y* and a binary value *d* ∈ {North, South} that determines whether 0 ≤ *ϕ* < *π* or *π* ≤ *ϕ* < 2*π*.

We assume that the rat only switches between North and South at the ends of the track. Because discontinuities in the functions lead to large Δ-values, slow functions *g*(*y*,*d*) should fulfill the continuity condition that *g*(0,North) = *g*(0,South) and *g*(*L_y_*,North) = *g*(*L_y_*,South). This means that the configuration space has the topology of a circle, where one half of the circle represents all positions with the rat facing North and the other half the positions with the rat facing South. It is thus convenient to introduce a different variable *ξ* ∈ [0,2*L_y_*] that labels the configurations in the following way:

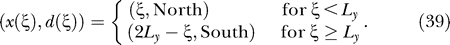



The topology of the configuration space is then captured by cyclic boundary conditions for the functions *g*(*ξ*).

For simplicity, we assume that there are no preferred positions or head directions, i.e., that both the variance of the velocity 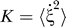 and the probability distribution *p*(*ξ*) is independent of *ξ*. The equation for the optimal function is then given by

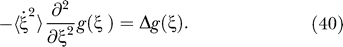



The solutions that satisfy the cyclic boundary condition and their Δ-values are given by

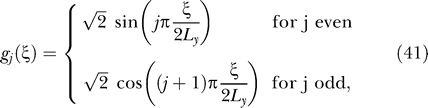


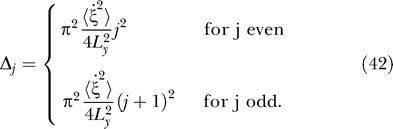



Note that there are always two functions with the same Δ-value. Theoretically, any linear combination of these functions has the same Δ-value and is thus also a possible solution. In the simulation, this degeneracy does not occur, because mid-track turns do occur occasionally, so those functions that are head-direction–dependent on mid-track (i.e., even *j*) will have higher Δ-values than theoretically predicted. This avoids mixed solutions and changes the order of the functions when ordered by slowness.

**Figure 6:**
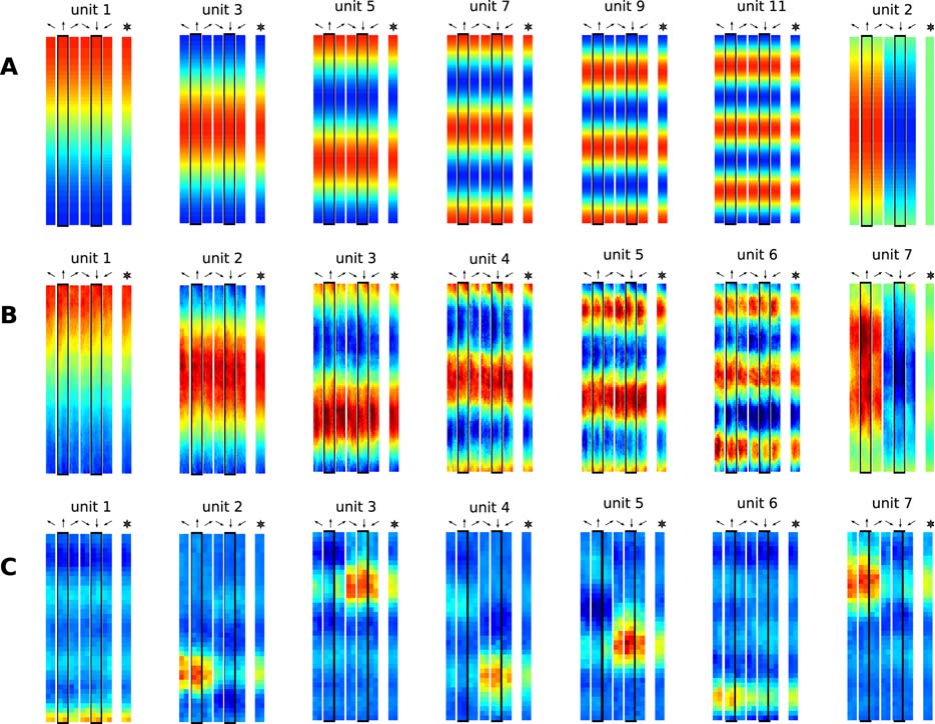
Theoretical Predictions and Simulation Results for the Linear Track Head directions are indicated by arrows, orientation averages are indicated by superimposed arrows, and principal directions (North, South) are emphasized with a dark border. Blue color denotes low activity, green intermediate activity, and red high activity. (A) Theoretical predictions. (B) Spatial firing maps of the first (i.e., slowest) seven out of ten SFA output units. Units 1–6 are mostly head-direction invariant, whereas unit 7 responds differently to North and South views. Two out of the three remaining units are also head-direction invariant. (C) Spatial firing maps of the first (i.e., most kurtotic) seven out of ten ICA output units. All units are localized in space, and most of them are only active for either North or South views closely resembling place fields recorded from rats in linear track experiments.

Figure 6A shows seven of the theoretically predicted functions *g_j_*, reordered such that they match the experimental results.

#### Simulation results for the linear track.

For simulations in the linear track, we use the more realistic movement paradigm similar to the open field experiment from the section Simulation results for the restricted head movement paradigm. A similar relative speed is assumed (*v_rel_* = 26), and sparse coding in the last layer is performed with ICA.

Figure 6B and 6C shows the simulation results for the linear track. The spatial firing maps of the seven slowest SFA outputs out of ten are shown in Figure 6B. Units 1–6 are mostly head-direction–invariant (*η_ϕ_* ≤ 0.1), and code for spatial position in the form of sine waves with frequencies of 1/2, 1, 3/2, 2, 5/2, and 3, as theoretically predicted. Units 7–10 (latter three not shown) code for position and orientation. At track ends, where most rotation occurs, all units are head-direction–invariant, and the spatial modulation is compressed due to slower mean translational speeds compared with mid-track (cf. the section Qualitative behavior of the solutions for inhomogeneous movement statistics). As expected, none of these units are localized in space or orientation.

The spatial firing maps of the first seven out of ten ICA outputs for different head directions are shown in Figure 6C. Units 1 and 6 are only active at the southern track end, independent of head direction. Units 9 and 10 (not shown) are active on mid-track and mostly independent of head direction (*η_ϕ_* ≤ 0.1). The other six units are localized in the joint position–head-direction space, meaning that they fire only at specific positions on the track when the rat faces a specific direction. These results are similar to place-cell recordings from rats in linear tracks where most cells only fire when the rat moves in one direction [33].

Changing the movement pattern to yield much higher or much lower mean relative rotational speeds can lead to very different results resembling those presented earlier for the open field, namely head-direction cells and head-direction–invariant place cells.

### Model Parameters

Although most of the parameters in our model (i.e., all the weights in the SFA and ICA steps) are learned in an unsupervised manner, a number of parameters were chosen by hand. These parameters include the input picture size, receptive field sizes, receptive field positions, and overlaps in all layers, the room shape, and textures, the expansion function space, number of layers, choice of sparsification algorithm, movement pattern, FOV, and number of training steps. We cannot explore the entire parameter space here and show instead that the model performance is very robust with respect to most of these parameters. The fact that the simulation results presented are very similar to the analytical solutions also indicates that the results presented are generic and not a mere artifact of a specific parameter set. The most interesting parameters are discussed in the following.

#### Image resolution.

We use high-resolution input pictures of 40 by 320 RGB pixels, showing the capability of the model to handle high-dimensional sensory data. However, it could be argued that the rat's vision is rather blurred and has little color sensitivity. We find that smaller and/or grayscale input pictures yield similar results, which degrade only below a dimensionality of a few hundred input pixels.

#### Field of view.

The model's FOV has been modeled to represent the 320° of a rat's FOV. Smaller FOVs down to 60° still reproduce our results, and especially rotation invariance is not an effect of a large FOV. However, the views have to contain enough visual information in order to fulfill the one-to-one correspondence between stimulus and oriospatial configuration.

#### Receptive fields.

The receptive fields are restricted to about 100 input dimensions (before quadratic expansion) due to computational limitations. Larger receptive fields tend to yield better solutions, since the available total function space increases. Position and overlap of receptive fields have been varied to some extent but have no noticeable impact on the result unless too many of the inputs are discarded.

#### Room shape.

The room shape has a strong impact on the SFA solutions, which can be predicted analytically. We show here only results from convex rooms, but experiments with radial mazes and multiple rooms have been performed and these results are similar to experimental data, too. Choice of specific textures was irrelevant for the model's performance except when multiple walls are textured with similar or identical textures, which leads to degraded results due to visual ambiguities. For small FOV values and symmetrical environments, the model's representations become symmetrical as well.

#### Nonlinear expansion.

The expansion function space was chosen as all monomials up to degree 2, but alternative function spaces like linear random mixtures passed through sigmoidals with different offsets were successful, too. However, the size of the function space is limited by computational constraints and monomials have proven to be particularly efficient. Even a linear function space is sufficient to generate a subset of the theoretically predicted results in some cases. The head-direction cell simulations reproduce seven out of eight optimal SFA solutions in the linear case and with a 320° FOV. In a linear place-cell simulation, only every second optimal SFA solution was found, and most of the ICA representations had two or more separate peaks. Simulations with a linear function space yield the theoretically predicted results only for a large FOV.

#### Number of layers.

The number of layers is determined by receptive field sizes and overlaps. An increased number of layers also increases the function space and can thus improve performance. We did not see any effect of overfitting for up to two more SFA layers. Additional top layers simply reproduced the output of earlier layers.

#### Sparse coding algorithm.

As for the choice of the sparse coding algorithm, we found no qualitative difference for different techniques including CuBICA, fastICA, CL, or just finding rotations of the SFA output with maximal kurtosis [27].

#### Movement statistics.

The choice of movement pattern has a clear impact on the optimal solutions of SFA. The theoretical analysis presented here can in principle predict the solutions for arbitrary movement patterns, but for the predictions presented here we made simplifying assumptions to obtain closed form solutions. In spite of these simplifications, the theoretical predictions are still close to the simulation results, e.g., in the section named Simulation results for the restricted head movement paradigm, where the head orientation is restricted to an angular range with respect to the direction of body motion.

In the movement paradigm for the spatial-view cells, the fixated point *X* changes smoothly over time without abrupt changes. If *X* instead changed seldom but abruptly, as by saccadic eye movement, similar representations as for smooth changes of *X* emerge (unpublished data), except that the SFA solutions need no longer be similar for adjacent viewpoints. However, in our simulations the similarity of the visual stimuli for adjacent view points often suffices for locally smooth responses.

#### Training set size.

More training steps result in a smoother sampling of the virtual reality environment and yield better approximations to the theoretical predictions. We found that a few laps crossing and spanning the whole room within 5,000 training samples were sufficient for the qualitative results. For too little training data and too few crossings of paths, an overfitting effect occurs resulting in a slowly varying activity of the outputs on the training path but not on other (test) paths.

## Discussion

We have presented a model for the formation of oriospatial cells based on the unsupervised learning principles of slowness and sparseness. The model is feed-forward, instantaneous, and purely sensory-driven. The architecture of the model is inspired by the hierarchical organization of the visual system and applies the identical learning rule, SFA, on all but the last layer, which performs sparse coding. Our results show that all major oriospatial cell types—place cells, head-direction cells, spatial-view cells, and to some extent even grid cells—can be learned with this approach. We have shown that this model is capable of extracting cognitive information such as an animal's position from complex high-dimensional visual stimuli, which we simulated as views in a virtual environment. The generated representations were coding specifically for some information (e.g., position) and were invariant to the others (e.g., head direction). These invariant representations are not explicitly built into the model but induced by the input statistics, which are in turn determined by the room shape and a specific movement paradigm. Nevertheless, the type of learned invariance can be influenced by a temporal adaptation of the learning rate. Control experiments show that the model performance is robust to noise and architectural details. This robustness is also supported by a general mathematical framework that allows exact analytical predictions of the system behavior at the top SFA level.

Our model comprises sensory processing stages that mimic parts of visual cortex and the hippocampal formation. The model layers cannot be exactly associated with specific brain areas, but we suggest some relations. The behavior of the lower two layers are primarily determined by the visual environment and mostly independent of the spatial movement pattern. In the simulations presented here, we trained the two lower layers only once and only adapted the higher layers for different environments and movement patterns. The first layer could be associated with V1 [1], the second layer with higher visual areas. Units in the third layer show a periodic non-localized spatial activity pattern (cf. Figure 4A and 4B), which strongly depends on the movement pattern and might be associated with grid cells in EC. However, two major differences between the SFA representations in the third layer and grid cells are notable. First, grid cells form a hexagonal grid, while the structure in the SFA representations depends on the shape of the room (rectangular rooms yield rectangular SFA patterns). Second, the lowest spatial frequency in the SFA representation is half the size of the simulated room, while the peak distances found in EC grid cells show intrinsic spatial scales that range from 39 cm to 73 cm [4].

The strong influence of room shape on the SFA results is due to the temporally global decorrelation and unit variance constraints in SFA. Thus, SFA requires a decorrelation of activities over arbitrarily long timescales, which might be difficult to achieve in a biologically plausible manner. We expect that a relaxation of these constraints to a limited time window leads to decorrelated representations only within the spatial range that is typically covered by the rat within this time window. This weakens the dependence of the results on the shape of the room and introduces an intrinsic spatial scale as found in EC. Preliminary results indicate that hexagonal activity patterns can emerge in such a system.

Depending on the movement statistics during learning, representations in the sparse coding layer resemble either place cells as found in hippocampal areas CA1 and CA3 or head-direction cells as found in many areas of the hippocampal formation or spatial-view cells as found in the hippocampal formation of monkeys. For the case of approximately uncorrelated body movement and head direction, the model learns either place or head-direction cells, depending on the relative speed of translation and rotation. For much quicker rotation than translation, the model develops orientation invariant place fields, while for much quicker translation than rotation the model develops position invariant head direction codes. In intermediate cases, e.g., for the linear track, mixed representations such as direction-dependent place fields emerge. Such mixed representations have also been reported in the subicular complex [34,35] and medial EC [12]. In the case of correlated body movement and head direction caused by elongated fixations of objects or positions, the model learns view-specific codes, similar to spatial-view cells in primates.

Although the model is capable of learning place cells and head-direction cells, if it learns on distinct adequate movement statistics, a model rat should obviously not have to traverse its environment once with low relative rotational speed to learn head-direction cells and once more with high relative rotational speed to learn place cells. How can both populations be trained with a single given input statistics? For this problem we have considered output from the rat's vestibular system as a possible solution. This system is essential for the oriospatial specificity of head-direction cells and place cells [36]. Other models like the well-established ring attractor model by Skaggs et al. [37] assume that the head direction system performs angular integration of body motion based on vestibular velocity signals. We hypothesize that these signals could also be used to influence the learning rate of two populations of cells that learn according to our model. One of these populations learns more strongly at periods with high relative translational speed (as signaled by the vestibular angular velocity signals), and the other adapts more strongly for low relative translational speed. The former should develop head-direction cell characteristics and the latter place cell characteristics. In our simulations, the model successfully learned both populations with the same input data, one population without LRA, and one population with reduced learning rate during quick rotations. Once the model has been trained, the vestibular acceleration signal is no longer needed for the model behavior. With LRA, the model neurons effectively learn on a different movement statistics, e.g., head-direction cells learn more strongly at times with relatively high translational speed. Nevertheless, if the real movement statistics contains very few episodes of relatively quick translation at all, the mechanism fails and head-direction cells cannot become position invariant. The principle of LRA is not limited to changing the effective relative rotational speed, as it can be adapted to reduce learning speed during episodes of quick changes of any feature, as long as some internal signal that is correlated with the change of the feature is available to control the LRA process. We expect that LRA could be used to concurrently learn spatial-view and place cells. This would require a faster change of gaze than in our view-cell simulations above. Then we expect that a population of cells trained without LRA develops place cell characteristics, whereas cells using LRA during episodes of fast fixation point changes develop spatial-view cell characteristics.

Our implementation of the slowness principle involves solving an eigenvalue problem and cannot be considered biologically plausible. However, more plausible implementations exist in the form of gradient-descent learning rules [22,38] and as a spike-timing–dependent plasticity rule [23]. The choice of ICA (and specifically our implementation based on CuBICA) to generate localized representations from nonlocalized codes might seem biologically unrealistic as well [but note 39], whereas a formulation in the form of nonlinear Hebbian learning [40] or CL seems more plausible. An in-depth discussion of this topic can be found in [27].

### Related Work

According to Redish's classification, our model is a *local view model,* for it “only depends on the local view to explain place- cell firing” [41]. Models of this class usually extract a number of features from sensory inputs in order to obtain a lower-dimensional representation that still carries information about spatial position in the environment but is invariant to everything else. Pure local view models do not comprise a path integration system and thus cannot fully explain oriospatial firing properties, e.g., in darkness. Pure path integration systems without external sensory input on the other hand accumulate errors, and hence a sensory coding mechanism, as proposed here, is necessary to complement any such model. Therefore, many models combine local view and path integration mechanisms [41,42], but here we focus only on local view models.

The model by Wyss et al. [43] is based on similar principles as our model. It applies a learning rule based on temporal stability to natural stimuli, some of which are obtained from a robot. The resulting spatial representations are localized, resembling hippocampal place fields. The learning rule involves local memory, and no explicit sparsification method is applied. The fact that the resulting representations are localized is somewhat surprising, since by itself temporal stability does not lead to localized representations [27]. This article does not investigate the influence of movement statistics on the learned representations.

The model by Sharp [44] assumes abstract sensory inputs and acquires a place code by CL, resulting in units that code for views with similar input features. Thus, this model is similar to our model's last layer performing sparsification. Similarly to our results, the degree of head-direction invariance depends on the movement statistics. Unlike our results, however, this is not due to the temporal structure of input views but to the relative density with which orientation or position are sampled.

The work by Fuhs et al. [45] uses realistic natural stimuli obtained by a robot and extracts “blobs” of uniform intensity with rectangular or oval shape from these images. Radial basis functions are tuned to blob parameters at specific views, and a CL scheme on these yields place cell-like representations. Our model agrees with their conclusion that rodents need no explicit object recognition in order to extract spatial information from natural visual stimuli.

The model by Brunel and Trullier [46] investigates the head-direction dependency of simulated place fields using abstract local views as inputs. A recurrent network learns with an unsupervised Hebbian rule to associate local views with each other, so that their intrinsically directional place cells can become head-direction–invariant for maze positions with many rotations. The article also conjectures that movement patterns determine head-direction dependence of place cells, which is consistent with our results.

The results by de Araujo et al. [47] suggest that the size of the rat's FOV is important for the distinction between spatial-view cells and place cells. With a large FOV (as for rats), the animal can see most landmarks from all orientations, while an animal with a small FOV (like a monkey) can only see a subset of all landmarks at each timepoint. We find no dependence of our results on the FOV size for values between 60° and 320° as long as the environment is rich enough (e.g., diverse textures, not a single cue card). Instead, our results suggest that differences in the movement statistics play a key role for establishing this difference.

To our knowledge, no prior model allows the learning of place cells, head-direction cells, and spatial-view cells with the same learning rule. Furthermore there are only a few models that allow clear theoretical predictions, learn oriospatial cells from (quasi) natural stimuli, and are based on a learning rule that is also known to model early visual processing well.

### Future Perspectives

Our simulated visual stimuli come from a virtual reality environment which is completely static during the training of the virtual rat. In this case, the slowest features are position, orientation, or view direction, as shown before. However, the assumption that the environment remains unchanged during oriospatial cell learning certainly does not hold for the real world. A more realistic environment will include other changing variables such as lighting direction, pitch and roll of the head, etc. The impact of these variables on the model representations depends on the timescale on which the variables change. For instance, the additional white noise in all SFA layers of the model is ignored since it varies much more quickly than position and orientation, but the direction of sunlight might become the slowest feature. Generally, the SFA solutions will depend on any variable whose timescale is equal to or slower than the position and orientation of the animal. After the sparse coding step, representations become not only localized in position and/or head direction but in the other variables as well. This behavior is not consistent with the definition of an ideal place or head-direction cell. However, many experiments show correlations of place-cell firing with nonspatial variables as well [41]. One particularly interesting instance of such a variable is “room identity.” If a rat experiences multiple environments, usually transitions between these will be seldom, i.e., the rat will more often turn and traverse a single room rather than switch rooms. In this case, room identity is encoded by the SFA outputs (unpublished data). For *n* rooms at most (*n* – 1) decorrelated SFA outputs can code for the room identity. The following outputs will then code for a joint representation of space and room identity. After sparse coding, many output units will fire in one room only (the less sparse ones in few rooms), and possibly in a completely unrelated fashion to their spatial firing patterns in another room. This behavior is consistent with the “remapping” phenomenon in place cells (e.g., [48]).

A great amount of work has been done investigating the impact of environmental manipulations on oriospatial cell firing in *known* rooms, e.g., shifts and rotations of landmarks relative to each other [41]. How would our model behave after such changes to the learned environment? Such transformations effectively lead to visual input stimuli outside the set of all possible views in the training environment. In this case, we expect the system's performance to deteriorate unless a new representation is learned, but more work is necessary to investigate this question.

Our approach predicts increasing slowness (i.e., decreasing Δ-values of firing rates) in the processing hierarchy between retina and hippocampus. Additionally, place cell and head-direction cell output should be significantly sparser than their inputs. Our main prediction is that changing movement statistics directly influences the invariance properties of oriospatial cells. For instance, an experiment in a linear track where the rat more often turns on mid-track should yield less head-direction–dependent place cells.

Our model is not limited to processing visual stimuli, as presented here, but can integrate other modalities as well. The integration of olfactory cues, for example, might lead to even more accurate representations and possibly to an independence of the model of visual stimuli (simulated darkness).

Experimentally, the joint positional and orientational dependence of oriospatial cells is hard to measure due to the size of the three-dimensional parameter space, and even more so if the development over time is to be measured. Furthermore, precise data on movement trajectories is rare in the existing literature on oriospatial cells. Accordingly, little data is available to verify or falsify our prediction of how the brain's oriospatial codes depend on the movement statistics. As an alternative to determining the movement statistics in behavioral tasks, some work has been done on passive movement of rats, where the movement statistics is completely controlled by the experimenter (e.g. [49]), but these results might not be representative for voluntary motion [50]. Markus et al. find directional place fields in the center of a plus maze, although more rotations occur in the center of the maze than in the arms [11]. This could be a contradiction to our model, although the relative speed (which was not measured in [11]) not the frequency determines head-direction invariance in our model. Overall, the dependence of oriospatial cells on the animal's movement statistics as proposed here remains to be tested experimentally.

### Conclusion

We conclude that a purely sensory-driven unsupervised system can reproduce many properties of oriospatial cells in the rodent brain, including place cells, head-direction cells, spatial-view cells, and to some extent even grid cells. These different cell types can be modeled with the same system, and the output characteristics depend solely on the movement statistics of the virtual rat. Furthermore, we showed that the integration of vestibular acceleration information can be used to learn place cells and head-direction cells with the same movement statistics and thus at the same time.

## Supporting Information

Protocol S1 Proofs of Theorems for Optimization Problem 2 (98 KB PDF)

## Abbreviations

CL: competitive learning
EC: entorhinal cortex
FOV: field of view
ICA: Independent Component Analysis
LRA: learning rate adaption
SFA: Slow Feature Analysis
