# Supplementary material for: Slowness and Sparseness Lead to Place, Head-Direction, and Spatial-View Cells
Source: PLoS Comput Biol. 2007 Aug 31;3(8):e166. doi: 10.1371/journal.pcbi.0030166 (PMC1963505; doi:10.1371/journal.pcbi.0030166)
Supplement: Protocol S1 — (98 KB PDF) [file pcbi.0030166.sd001.pdf]

Protocol S1 for “Slowness and Sparseness Lead to Place, Head-Direction, and Spatial-View Cells”

Mathias Franzius, Henning Sprekeler, Laurenz Wiskott

Institute for Theoretical Biology, Humboldt-Universität zu Berlin.
{m.franzius, h.sprekeler, l.wiskott}@biologie.hu-berlin.de

Optimization problem 2

Given a function space $\mathcal{F}$ on a configuration space V, which is sampled with probability density $p_{\mathbf{s},\mathbf{v}}(\mathbf{s},\mathbf{v})$, find a set of J functions $g_j(\mathbf{s}) \in \mathcal{F}$ that

$$\text{minimize } \Delta(\mathrm{g_j}) := \left\langle (\text{Ñ}g_j(\mathbf{s}) \cdot \mathbf{v})^2 \right\rangle_{\mathbf{s},\mathbf{v}} \tag{9}$$

under the constraints

$$\left\langle g_j(\mathbf{s}) \right\rangle_{\mathbf{s}} = 0 \qquad \text{(zero mean)}, \tag{10}$$

$$\left\langle g_j(\mathbf{s})^2 \right\rangle_{\mathbf{s}} = 1 \qquad \text{(unit variance)}, \tag{11}$$

$$\forall i < j : \left\langle g_i(\mathbf{s}) g_j(\mathbf{s}) \right\rangle_{\mathbf{s}} = 0 \qquad \text{(decorrelation and order)}. \tag{12}$$

According to the method of Lagrange multipliers a necessary condition for the solutions of this optimization problem is that the objective function

$$\Psi(g_j) = \frac{1}{2}\Delta(g_j) - \lambda_{j0} \left\langle g_j(\mathbf{s}) \right\rangle_{\mathbf{s}} - \frac{1}{2}\lambda_{jj} \left\langle g_j(\mathbf{s})^2 \right\rangle_{\mathbf{s}} - \sum_{i<j} \lambda_{ji} \left\langle g_i(\mathbf{s}) g_j(\mathbf{s}) \right\rangle_{\mathbf{s}}. \tag{13}$$

is stationary. This leads to

Theorem 1

For a particular choice of the parameters λ_{ij}, the solutions g_j of optimization problem 2 obey the Euler-Lagrange equation

$$\mathcal{D} g_j(\mathbf{s}) - \lambda_{j0} - \lambda_{jj} g_j(\mathbf{s}) - \sum_{i<j} \lambda_{ji} g_i(\mathbf{s}) = 0 \tag{15}$$

with the boundary condition

$$\mathbf{n}(\mathbf{s})^T \mathbf{K}(\mathbf{s}) \nabla g_j(\mathbf{s}) = 0 \qquad \text{for } \mathbf{s} \in \partial V. \tag{16}$$

Here, the partial differential operator $\mathcal{D}$ is defined as

$$\mathcal{D} := -\frac{1}{p_{\mathbf{s}}(\mathbf{s})} \nabla \cdot p_{\mathbf{s}}(\mathbf{s}) \mathbf{K}(\mathbf{s}) \nabla \tag{17}$$

and $\mathbf{n}(\mathbf{s})$ is the unit normal vector on the boundary ∂V of the configuration space V.

Proof: We are looking for stationary points of the objective function (13). As the function space is infinite-dimensional, this requires variational calculus, which can be illustrated by means of an expansion in the spirit of a Taylor expansion. Let us assume, we knew the function g_j that optimizes the objective function Ψ. The effect of a small change δg of g_j on the objective function Ψ can be written as

$$\Psi(g_j + \delta g_j) - \Psi(g_j) = \int \frac{\delta \Psi}{\delta g}(\mathbf{s}) \delta g(\mathbf{s}) d\mathbf{s} + ..., \tag{S1}$$

where the ellipses stand for higher order terms in δg. The function $\frac{\delta \Psi}{\delta g}$ is the *variational derivative* of the functional Ψ and usually depends on the configuration, the optimal function g_j, and possibly derivatives of g_j. Its analogue in finite-dimensional calculus is the gradient.

We now derive an expression for the variational derivative of the objective function (13). To keep the calculations tidy, we split the objective in two parts and omit the dependence on the configuration $\mathbf{s}$

$$\Psi(g_j) =: \frac{1}{2}\Delta(g_j) - \tilde{\Psi}(g_j). \tag{S2}$$

The expansion of $\tilde{\Psi}$ is straightforward:

$$\tilde{\Psi}(g_j + \delta g) - \tilde{\Psi}(g_j) = \left\langle \delta g \left[\lambda_{jo} + \lambda_{jj} g_j + \sum_{i<j} \lambda_{ji} g_i \right] \right\rangle_{\mathbf{s}} + \dots \tag{S3}$$

$$= \int \delta g \; p_{\mathbf{s}} \left[\lambda_{j0} + \lambda_{jj} g_j + \sum_{i<j} \lambda_{ji} g_i \right] d\mathbf{s} + ... \tag{S4}$$

For the expansion of $\Delta(g_j)$ we first simplify the expression by carrying out the velocity integration and using the velocity tensor $\mathbf{K}$:

$$\Delta(g_j) \overset{(9)}{=} \left\langle \nabla g_j^T \mathbf{v}\mathbf{v}^T \nabla g_j \right\rangle_{\mathbf{s},\mathbf{v}} = \left\langle \nabla g_j^T \left\langle \mathbf{v}\mathbf{v}^T \right\rangle_{\mathbf{v}|\mathbf{s}} \nabla g_j \right\rangle_{\mathbf{s}} \overset{(14)}{=} \left\langle \nabla g_j^T \mathbf{K} \nabla g_j \right\rangle_{\mathbf{s}}. \tag{S5}$$

We can now expand $\Delta(g_j)$ as follows:

$$\frac{1}{2}\Delta(g_j + \delta_g) - \frac{1}{2}\Delta(g_j) \overset{(S5)}{=} \frac{1}{2}\Delta\left\langle \nabla(g_j + \delta g)^T \mathbf{K} \nabla(g_j + \delta g) \right\rangle_{\mathbf{s}} - \frac{1}{2}\left\langle \nabla g_j^T \mathbf{K} \nabla g_j \right\rangle_{\mathbf{s}} \tag{S6}$$

$$= \frac{1}{2}\left\langle \nabla g_j^T \mathbf{K} \nabla \delta g + \nabla \delta g^T \mathbf{K} \nabla g_j \right\rangle_{\mathbf{s}} + \dots \tag{S7}$$

$$= \left\langle \nabla \delta g^T \mathbf{K} \nabla g_j \right\rangle_{\mathbf{s}} + \dots \quad \text{(since } \mathbf{K} \text{ is symmetric)} \tag{S8}$$

$$\overset{(8)}{=} \int p_{\mathbf{s}} \nabla \delta g^T \mathbf{K} \nabla g_j \; d\mathbf{s} \tag{S9}$$

$$= \int \nabla \cdot \left[\delta g \; p_{\mathbf{s}} \mathbf{K} \nabla g_j \right] d\mathbf{s} - \int \delta g \; \nabla \cdot (p_{\mathbf{s}} \mathbf{K} \nabla g_j) \; d\mathbf{s} + \dots \tag{S10}$$

$$= \int_{\partial V} \delta g \; p_{\mathrm{s}} \mathbf{n}^T \mathbf{K} \nabla g_j \; dA - \int \delta g \nabla \cdot (p_{\mathrm{s}} \mathbf{K} \nabla g_j) \; d\mathbf{s} + \ldots \quad \text{(Gauss' theorem)} \tag{S11}$$

$$\overset{(17)}{=} \int_{\partial V} \delta g \; p_{\mathrm{s}} \mathbf{n}^T \mathbf{K} \nabla g_j \; dA + \int \delta g \; p_{\mathrm{s}} (\mathcal{D} g_j) \; d\mathbf{s} + \ldots \; . \tag{S12}$$

Here, dA is an infinitesimal surface element of the boundary ∂V of V and $\mathbf{n}$ is the normal vector on dA. To get the expansion of the full objective function, we add (S4) and (S12):

$$\Psi(g_j + \delta g) - \Psi(g_j) = \int_{\partial V} \delta g \; p_{\mathrm{s}} \mathbf{n}^T \mathbf{K} \nabla g_j \; dA + \int \delta g \; p_{\mathrm{s}} (\mathcal{D} g_j - \lambda_{j0} - \lambda_{jj} g_j - \sum_{i<j} \lambda_{ji} g_i) \; d\mathbf{s} + \ldots \; . \tag{S13}$$

In analogy to the finite-dimensional case, g_j can only be an optimum of the objective function Ψ if any small change δg leaves the objective unchanged up to linear order. As we employ a Lagrange multiplier ansatz, we have an unrestricted optimization problem, so we are free in choosing δg. From this it is clear that the right hand side of (S13) can only vanish if the integrands of both the boundary and the volume integral vanish separately. This leaves us with the differential equation (15) and the boundary condition (16).

Theorem 2

Let $\mathcal{F}_b \subset \mathcal{F}$ be the space of functions that obey the boundary condition (16)*. Then $\mathcal{D}$ is self-adjoint on $\mathcal{F}_b$ with respect to the scalar product*

$$(f, g) := \langle f(\mathbf{s})\, g(\mathbf{s}) \rangle_{\mathbf{s}}, \tag{18}$$

i.e.

$$\forall f, g \in \mathcal{F}_b: \ (\mathcal{D}f, g) = (f, \mathcal{D}g). \tag{19}$$

Proof: The proof can be carried out in a direct fashion. Again, we omit the explicit dependence on $\mathbf{s}$.

$$\left(f, \mathcal{D}g\right) \overset{(8,17,18)}{=} -\int p_{\mathrm{s}} f \frac{1}{p_{\mathrm{s}}} \nabla \cdot p_{\mathrm{s}} \mathbf{K} \nabla g \; d\mathbf{s} \tag{S14}$$

$$= -\int \nabla \cdot \left[p_{\mathrm{s}} f \; \mathbf{K} \nabla g \right] d\mathbf{s} + \int p_{\mathrm{s}} \nabla f^T \mathbf{K} \nabla g \; d\mathbf{s} \tag{S15}$$

$$= -\int_{\partial V} p_{\mathrm{s}} f \; \underbrace{\mathbf{n}^T \mathbf{K} \nabla g}_{\overset{(16)}{=}\,0} \; dA + \int p_{\mathrm{s}} \nabla f^T \mathbf{K} \nabla g \; d\mathbf{s} \quad \text{(Gauss' theorem)} \tag{S16}$$

$$\overset{(16)}{=} \int p_{\mathrm{s}} \nabla f^T \mathbf{K} \nabla g \; d\mathbf{s} \tag{S17}$$

$$= \int p_{\mathrm{s}} \nabla g^T \mathbf{K} \nabla f \; \mathrm{d}\mathbf{s} \quad \text{(since } \mathbf{K} \text{ is symmetric)} \tag{S18}$$

$$\overset{(S14-S16)}{=} \left(\mathcal{D}f, g\right). \tag{S19}$$

Theorem 3

Apart from the constant function, which is always an eigenfunction, the (adequately normalized) eigenfunctions $f_j \in \mathcal{F}_b$ of the operator $\mathcal{D}$ fulfill the constraints (10)-(12*).*

Proof :

Zero mean: It is obvious that the constant function $f_0 = 1$ is always an eigenfunction of $\mathcal{D}$ for eigenvalue 0. As all other eigenfunctions are orthogonal to f_0, they must have zero mean: $\left(f_0, f_j\right) = \left\langle f_j \right\rangle_{\mathbf{s}} = 0 \quad \forall j \neq 0$.

Decorrelation: For mean-free functions f and g the scalar product $\left(f, g\right)$ is their covariance. The orthogonality of the eigenfunctions is thus equivalent to decorrelation.

Unit variance: Unit variance can easily be achieved by renormalizing the eigenfunctions such that $\left(f, f\right) = \left\langle f^2 \right\rangle_{\mathbf{s}} = 1$.

Theorem 4

The Δ-value of the normalized eigenfunctions f_j is given by their eigenvalue Δ_j.

Proof:

$$\Delta(f_j) \overset{(S5,S14-S18)}{=} \left(f_j, \mathcal{D}f_j\right) = \left(f_j, \Delta_j f_j\right) = \Delta_j \underbrace{\left(f_j, f_j\right)}_{=1} = \Delta_j \,. \quad \text{(S20)}$$

Theorem 5

The J eigenfunctions with the smallest eigenvalues $\Delta_j \neq 0$ are a solution of optimization problem 2.

Proof : Without loss of generality we assume that the eigenfunctions f_j are ordered by increasing eigenvalue, starting with the constant $f_0 = 0$. There are no negative eigenvalues, because the eigenvalue is the Δ-value of the eigenfunction, which can only be positive
by definition. According to Theorem 1, the optimal responses g_j obey the boundary condition (16) and are thus elements of the subspace $\mathcal{F}_b \subset \mathcal{F}$ defined in Theorem 2. Because of the completeness of the eigenfunctions on $\mathcal{F}_b$ we can do the expansion

$$g_j = \sum_{k=1}^{\infty} \alpha_{jk} f_k \quad \text{(S21)}$$

where we may omit f_0 because of the zero mean constraint. We can now prove by complete induction that $g_j = f_j$ solves the optimization problem.

Basis (j=1): Inserting g_1 into eqn. (15) we find

$$0 = \mathcal{D}g_1 - \lambda_{10} - \lambda_{11} g_1 \quad \text{(S22)}$$

$$= -\lambda_{10} + \sum_{k=1}^{\infty} \alpha_{1\kappa} (\Delta_k - \lambda_{11}) f_k \quad \text{(S23)}$$

$$\Rightarrow \begin{array}{ll} & \lambda_{10} = 1 \\ \wedge & (\alpha_{1k} = 0 \;\vee\; \Delta_k = \lambda_{11}) \;\; \forall k, \end{array} \quad \text{(S24)}$$

because f_k and the constant are linearly independent and (S22) must be fulfilled for all $\mathbf{s}$. (S24) implies that the optimal response g_1 must be an eigenfunction of $\mathcal{D}$. As the Δ-value of the eigenfunctions is given by their eigenvalue, it is obviously optimal to chose $g_1 = f_1$. Note that although this choice is optimal, it is not necessarily unique, since there may be several eigenfunctions with the same eigenvalue. In this case any linear combination of these functions is also optimal.

Induction step: Given that $g_i = f_i$ for $i < j$, we prove that $g_j = f_j$ is optimal. Because of the orthonormality of the eigenfunctions the decorrelation constraint (12) yields

$$0 \overset{(12)}{=} \left\langle g_i g_j \right\rangle_{\mathbf{s}} = \left(f_i, \sum_{k=1}^{\infty} \alpha_{jk} f_k \right) = \alpha_{ji} \qquad \forall i < j \; . \tag{S25}$$

Again inserting the expansion (S21) into eqn. (15) yields

$$0 \overset{(15,S21)}{=} (\mathcal{D} - \lambda_{jj}) \sum_{k=1}^{\infty} \alpha_{jk} f_k - \lambda_{j0} - \sum_{i<j} \lambda_{ji} f_i \tag{S26}$$

$$\overset{(S25)}{=} (\mathcal{D} - \lambda_{jj}) \sum_{k=j}^{\infty} \alpha_{jk} f_k - \lambda_{j0} - \sum_{i<j} \lambda_{ji} f_i \tag{S27}$$

$$\overset{(20)}{=} \sum_{k=j}^{\infty} (\Delta_k - \lambda_{jj}) \alpha_{jk} f_k - \lambda_{j0} - \sum_{i<j} \lambda_{ji} f_i \tag{S28}$$

$$\begin{aligned} & & \lambda_{j0} &= 0 & & \\ \Rightarrow & \quad \wedge & \lambda_{ji} &= 0 & & \forall i < j \\ & \quad \wedge & (\alpha_{jk} = 0 &\vee \Delta_k = \lambda_{jj}) & & \forall k \geq j \end{aligned} \tag{S29}$$

because the eigenfunctions f_i are linearly independent. The conditions (S29) can only be fulfilled if g_j is an eigenfunction of $\mathcal{D}$. Because of Theorem 4 an optimal choice for minimizing the Δ-value without violating the decorrelation constraint is $g_j = f_j$.
